# Parameters Affecting Continuous *In Vitro* Culture of Treponema pallidum Strains

**DOI:** 10.1128/mBio.03536-20

**Published:** 2021-02-23

**Authors:** Diane G. Edmondson, Bridget D. DeLay, Lindsay E. Kowis, Steven J. Norris

**Affiliations:** a Department of Pathology and Laboratory Medicine, University of Texas Health Science Center at Houston, Houston, Texas, USA; b Department of Microbiology and Molecular Genetics, McGovern Medical School, University of Texas Health Science Center at Houston, Houston, Texas, USA; NIAID, NIH

**Keywords:** *Treponema pallidum*, bejel, culture, epithelial cells, growth requirements, oxygen, physiology, syphilis

## Abstract

The bacterium that causes syphilis, Treponema pallidum subsp. *pallidum*, has now been cultured *in vitro* continuously for periods exceeding 3 years using a system consisting of coculture with Sf1Ep rabbit epithelial cells in TpCM-2 medium and a low-oxygen environment. In addition, long-term culture of several other syphilis isolates (SS14, Mexico A, UW231B, and UW249B) and the T. pallidum subsp. *endemicum* Bosnia A strain has been achieved. During *in vitro* passage, T. pallidum subsp. *pallidum* exhibited a typical bacterial growth curve with logarithmic and stationary phases. Sf1Ep cells are required for sustained growth and motility; however, high initial Sf1Ep cell numbers resulted in reduced multiplication and survival. Use of Eagle’s minimal essential medium as the basal medium was not effective in sustaining growth of T. pallidum subsp. *pallidum* beyond the first passage, whereas CMRL 1066 or M199 supported long-term culture, confirming that additional nutrients present in these more complex basal media are required for long-term culture. T. pallidum subsp. *pallidum* growth was dependent upon the presence of fetal bovine serum, with 20% (vol/vol) being the optimal concentration. Omission of reactive oxygen species scavengers dithiothreitol, d-mannitol, or l-histidine did not dramatically affect survival or growth. Additionally, T. pallidum subsp. *pallidum* can be successfully cultured in a Brewer jar instead of a specialized low-oxygen incubator. Phosphomycin or amphotericin B can be added to the medium to aid in the prevention of bacterial or fungal contamination, respectively. These results help define the parameters of the T. pallidum subsp. *pallidum* culture system that are required for sustained, long-term survival and multiplication.

## INTRODUCTION

The species Treponema pallidum, first described in 1905 (1, 2), is subdivided into closely related subspecies that cause syphilis (T. pallidum subsp. *pallidum*), yaws and nonhuman primate infections (T. pallidum subsp. *pertenue*), and bejel/endemic syphilis (T. pallidum subsp. *endemicum*) (3–7). Other spirochetes in this group cause pinta (*T. carateum*) and venereal spirochetosis of rabbits and hares (*T. paraluiscuniculi*; proposed name change to “*T. paraluisleporidarum*”) (7, 8). Members of this group of organisms are morphologically indistinguishable and have nearly identical genomes, with the T. pallidum subspecies sharing >99.78% sequence identity with one another and ∼99.2% identity with *T. paraluiscuniculi* (9, 10). Attempts to culture the T. pallidum-related pathogens had been unsuccessful for over 100 years despite concerted efforts (11–14), necessitating the maintenance of strains through serial infection of rabbits or other mammals (15, 16). This fastidious nature appears to be associated with an extreme adaptation to life within mammalian tissue, accompanied by genome reduction and the substantial loss of metabolic and biosynthetic capabilities (3, 14, 17, 18). Studies in the mid-1900s revealed the microaerophilic nature of T. pallidum, the dependence on serum components, and prolonged survival in the presence of mammalian cells; however, reproducible multiplication of the organism *in vitro* was not observed (reviewed in references 3, 14, 17, 19).

In 1981, Fieldsteel et al. (20) reported the successful short-term *in vitro* culture of T. pallidum subsp. *pallidum*, resulting in up to 100-fold multiplication over a period of 12 to 18 days (6 to 7 doublings). This system utilized coincubation of the Nichols strain of T. pallidum subsp. *pallidum* with Sf1Ep cottontail rabbit epithelial cells in a modified tissue culture medium (called basal reduced medium-modified [BRMM]) in a microaerobic environment containing 1.5% oxygen and 5% CO_2_ (19, 20). Cox (19) later amended this medium with additional compounds and enzymes, resulting in T. pallidum cultivation medium (herein called TpCM-1). Replication of T. pallidum subsp. *pallidum* Nichols and other strains in this system was highly reproducible, but attempts to extend survival and multiplication beyond a few weeks were unsuccessful (14, 19, 21).

In 2018, the first successful long-term cultivation of T. pallidum subsp. *pallidum* was reported (22). This study utilized the Fieldsteel et al. system with the introduction of an altered medium, TpCM-2; the principal modification was the replacement of the basal medium Eagle’s minimal essential medium (Eagle’s MEM) with a more complex tissue culture medium, CMRL 1066. With this alteration, multiplication, morphologic integrity, and full viability and infectivity of T. pallidum subsp. *pallidum* Nichols were maintained for over 6 months by serial passage; the more recent T. pallidum subsp. *pallidum* isolates UW231B and UW249B were also successfully cultured for shorter periods (22). In the current study, we report the continuous culture of T. pallidum subsp. *pallidum* Nichols for periods exceeding 3 years and the long-term culture of several other strains. We have also further defined the growth requirements of T. pallidum by examining the effects of parameters such as the mammalian cell requirement, medium composition, and oxygen concentration on survival and multiplication. These findings indicate that the T. pallidum cultivation system is robust and highly reproducible and can facilitate study of this highly fastidious organism.

## RESULTS

### Continuous culture of T. pallidum.

**FIG 1:**
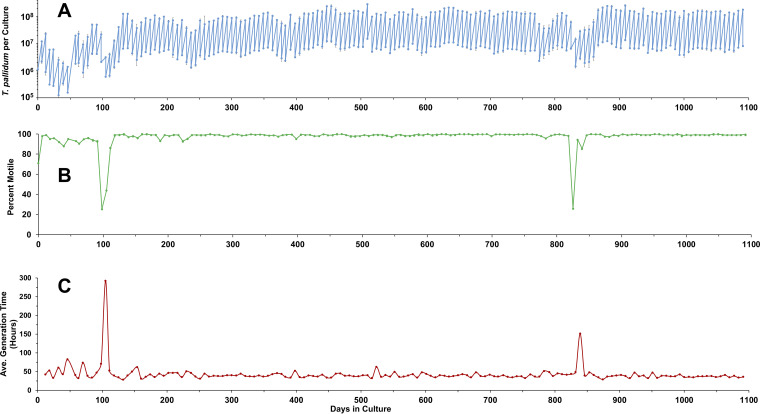
Long-term *in vitro* culture of T. pallidum, as exemplified by a lineage of T. pallidum subsp. *pallidum* Nichols maintained continuously for over 3 years. (A) Sawtooth plot showing the numbers of T. pallidum per culture and the number transferred to new cultures at each time point. Results represent the mean ± SEM for three biological replicates. (B) Percent of motile organisms, expressed as mean of three biological replicates. (C) Average generation time in hours for each time point. Decreases in T. pallidum per culture and percent motility and corresponding increases in generation time correspond to times of decreased growth and viability related to Sf1Ep cell culture status or medium composition issues (see the text). Representative of two long-term experiments (Table 1).

In an earlier article (22), we reported the continuous culture of the Nichols strain of T. pallidum subsp. *pallidum* for up to 189 days using a tissue culture system with TpCM-2 medium and incubation at 1.5% O_2_ and 5% CO_2_ at 34°C. Cultures of the more recent T. pallidum subsp. *pallidum* isolates UW231B and UW249B had been maintained for 70 and 40 days, respectively. At the time of this writing, two T. pallidum subsp. *pallidum* Nichols cultures have been passaged continuously for over 3 years using the same culture system (Table 1; Fig. 1). Cultures of strains UW231B and UW249B have also now been maintained for >200 days. In addition, the syphilis isolates T. pallidum subsp. *pallidum* SS14 and Mexico A and the bejel isolate T. pallidum subsp. *endemicum* Bosnia A have been cultured *in vitro* successfully for the first time (Table 1). Attempts to obtain consistent *in vitro* multiplication of the T. pallidum subsp. *pertenue* strains Gauthier and Samoa F have thus far been unsuccessful, despite retention of motility for up to 14 days (data not shown).

**TABLE 1:** Information on continuous culture of T. pallidum subspecies and strains

T. pallidum subspecies and strain	T. pallidum cluster	Culture status	Days in culture	Passage no.	Cumulative no. of generations	Mean^*a*^ generation time ± SE (h)	Minimum^*b*^ generation time (h)
T. pallidum subsp. *pallidum*							
Nichols	Nichols	Ongoing	1,110	157	634	41.5 ± 0.5	30.0
Nichols	Nichols	Ongoing	1,091	156	656	40.1 ± 0.5	28.6
SS14	SS14	Completed	203	29	88	56.6 ± 1.8	44.1
Mexico A	SS14	Ongoing	203	29	103	47.5 ± 1.6	36.8
UW231B	SS14	Completed	203	29	88	56.6 ± 1.8	42.8
UW249B	SS14	Completed	240	34	110	54.4 ± 1.7	41.8
T. pallidum subsp. *endemicum*							
Bosnia A		Ongoing	133	18	58	53.1 ± 2.0	41.0

a Outliers were removed from the T. pallidum subsp. *pallidum* Nichols cultures before mean calculations. Outliers were removed when we could identify a reason for the large change in generation time. These include one error in passage timing and instances where high passage number Sf1Ep cells could no longer support T. pallidum multiplication. Examples of these outliers are apparent in Fig. 1C.

b Minimum, lowest generation time obtained for a single passage.

As reported previously (22), T. pallidum cultures exhibit a highly consistent growth pattern when subcultured at 7-day intervals. Periods of reduced multiplication and viability have occurred (Fig. 1A and B) and appear to be related to either medium composition issues or the use of high-passage-number Sf1Ep cell cultures (which proliferate more rapidly). All T. pallidum strains examined exhibit a long generation time during *in vitro* culture, consistent with the estimated generation time of 32 to 35 h during experimental rabbit infection (23, 24). In our studies, T. pallidum subsp. *pallidum* Nichols had mean generation times of approximately 40.1 to 41.5 h, whereas the other strains had somewhat longer mean generation times of 47.5 to 56.8 h (Table 1). Values representing the minimum generation times are also provided as a measure of growth rates under optimal conditions. When we analyzed differences in mean generation times using the Student’s *t* test (see Table S1 in the supplemental material), we found that there was no significant difference between the generation times of the two long-term Nichols cultures. However, the Nichols cultures had significantly shorter generation times than all other strains tested. Likewise, the Mexico A strain had generation times significantly shorter than all other strains except Nichols. There was no significant difference in the mean generation times of the SS14, UW231B, UW249B, or Bosnia A strains. In all cases, the strains examined have retained the characteristic T. pallidum morphology and >90% motility, with the common occurrence of clumps of 3 to >10 organisms.

10.1128/mBio.03536-20.8 TABLE S1 *P* values for differences in generation times between T. pallidum strains. Download Table S1, PDF file, 0.2 MB. Copyright © 2021 Edmondson et al. 2021 Edmondson et al. https://creativecommons.org/licenses/by/4.0/ This content is distributed under the terms of the Creative Commons Attribution 4.0 International license.

A key feature of the *in vitro* culture system is the retention of full infectivity, as determined by lesion development following the intradermal inoculation of serial dilutions of cultures into rabbits. In the prior report (22), we had demonstrated retention of infectivity for at least 116 days of culture. In more recent experiments, T. pallidum subsp. *pallidum* cultures inoculated into rabbits on days 237, 399, and 557 of continuous *in vitro* culture have retained high infectivity, with median infectious doses ranging from 10 to 100 T. pallidum (Table 2).

**TABLE 2:** Retention of infectivity of *in vitro*-cultured T. pallidum subsp. *pallidum* Nichols^*a*^

T. pallidum dosage per site	Lesions/sites inoculated	Day of lesion development	Avg day of lesion development
237-Day culture			
1,000,000	4/4	4, 4, 5, 5	4.5
10,000	4/4	11, 11, 12, 12	11.5
100	4/4	19, 19, 22, 22	20
399-Day culture			
100,000	4/4	11, 10, 11, 11	10.8
10,000	4/4	13, 14, 14, 14	13.8
1,000	4/4	15, 15, 16, 19	16.3
100	2/4	22, 20	21
10	1/4	24	24
1	0/4		
557-Day culture			
100,000	4/4	9, 10, 9, 9	9.3
10,000	4/4	10, 11, 12, 13	11.5
1,000	4/4	13, 13, 13, 14	13.3
100	4/4	18, 20, 21, 23	20.5
10	4/4	22, 23, 22, 25	22.3

a Determined by intradermal inoculation of rabbits. Long-term cultures of T. pallidum subsp. *pallidum* Nichols initiated on 3 November 2017 were utilized as inoculum for the 237-day and 399-day infectivity studies, whereas another culture lineage initiated 20 October 2017 was used as the source of inoculum for the 557-day study.

### T. pallidum exhibits a standard bacterial growth curve.

**FIG 2:**
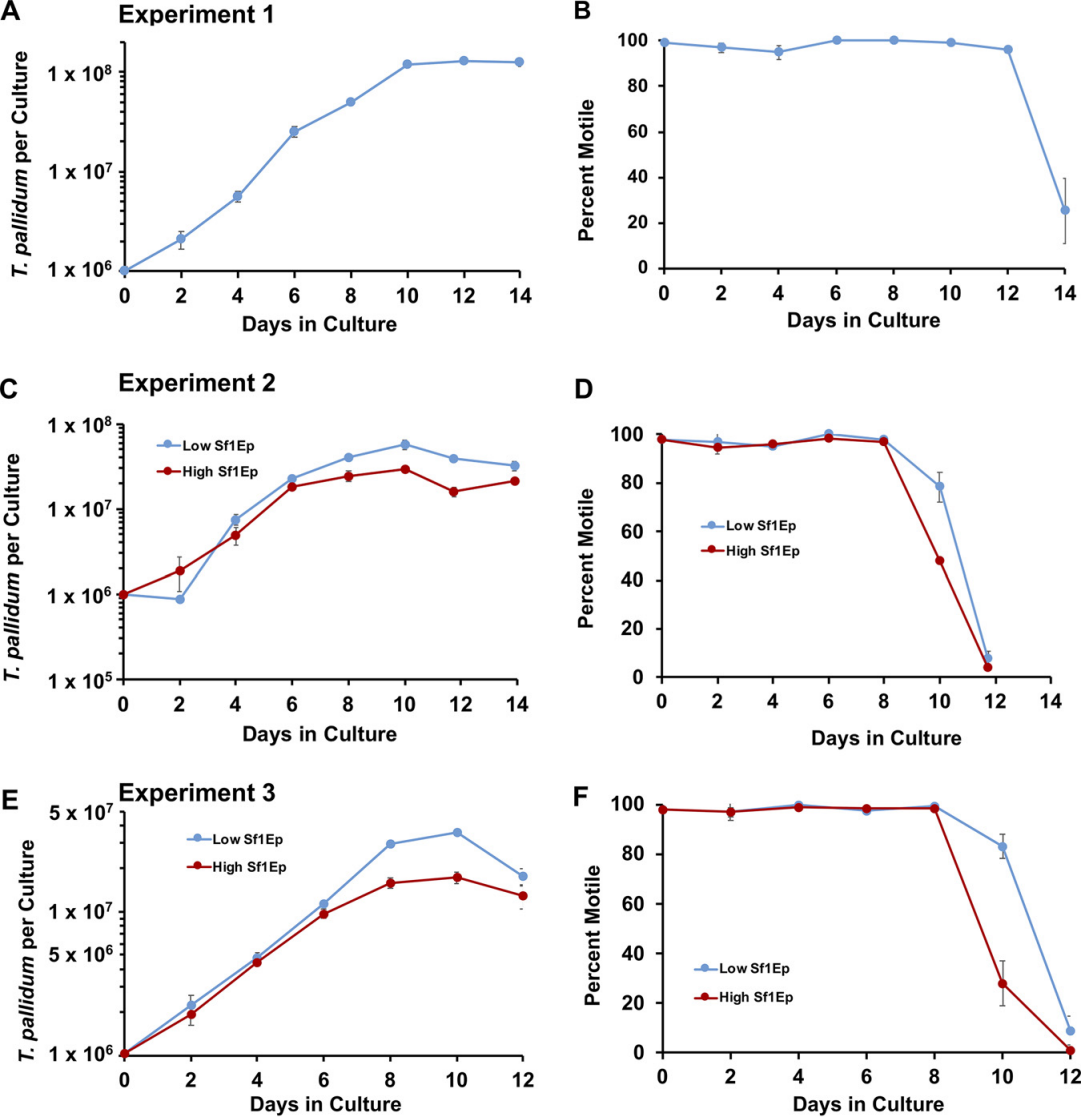
Growth curves of T. pallidum subsp. *pallidum* Nichols cultured in the Sf1Ep system with TpCM-2 medium. The changes in T. pallidum per culture and percent motility are shown for experiment 1 (A, B), experiment 2 (C, D), and experiment 3 (E, F). Experiment 1 consisted of a single condition in which 1 × 10^5^ Sf1Ep cells were added per culture. In experiments 2 and 3, parallel triplicate cultures were seeded with 3 × 10^4^ Sf1Ep cells (low inoculum) or 1 × 10^5^ Sf1Ep cells (high inoculum). The T. pallidum inoculum was adjusted to 1 × 10^6^ per culture in these experiments, so in experiments 2 and 3, the multiplicity of infection (MOI) was 33 and 10 in the low and high Sf1Ep concentration cultures, respectively. In each experiment and condition, three biological replicates were harvested at each time point; the results represent the mean ± SEM for these cultures.

Typically, T. pallidum cultured *in vitro* is passed every 7 days into new culture plates containing fresh TpCM-2 medium and newly plated Sf1Ep cottontail rabbit epithelial cells. To determine if this 7-day passage schedule is ideal for T. pallidum culture, a series of growth curves were conducted in which T. pallidum concentration and motility were monitored by harvesting parallel cultures at 2-day intervals for 14 days (Fig. 2). A consistent inoculum of 1 × 10^6^ T. pallidum per culture was utilized in these studies. In the first experiment, T. pallidum subsp. *pallidum* Nichols was added to 6-well cell culture plates containing the standard initial inoculum of 1 × 10^5^ Sf1Ep cells per well. The number of T. pallidum subsp. *pallidum* per well steadily increased over the first 6 to 10 days before plateauing (Fig. 2A, C, and E). Likewise, motility remained high for the first 6–10 days, before starting to drop by days 8 to 12 (Fig. 2B). Motility is a good indication of T. pallidum viability, so these results indicate that both growth and viability declined after 8 to 10 days of culture. Consistent with the decline in T. pallidum subsp. *pallidum* growth and motility, the medium color changed from pink to yellow around day 8 to 9, likely corresponding to changes in the redox potential, pH, and nutrient content of the medium, as noted in prior short-term culture studies (21). Overall, T. pallidum subsp. *pallidum* exhibits a typical growth curve with logarithmic and stationary stages; lag stages are only occasionally observed, most likely because cultures are routinely passaged at 7-day intervals when the spirochetes are still in the late logarithmic phase. T. pallidum subsp. *pallidum* viability is sustained for only a few days during the stationary phase.

Two additional experiments examined the effect of initial Sf1Ep cell concentration on T. pallidum subsp. *pallidum* Nichols survival and growth (Fig. 2C to F). A lower inoculum of Sf1Ep cells (3 × 10^4^) was compared with the standard inoculum of 1 × 10^5^ Sf1Ep cells per well. In each case, T. pallidum subsp. *pallidum* grown in wells with the lower inoculum of Sf1Ep cells reached a higher density at 10 days of *in vitro* culture than those grown in wells containing the standard, higher Sf1Ep inoculum (Fig. 2C and E). In these experiments, motility began to decrease after 8 days, with a more rapid drop in wells containing the higher inoculum of Sf1Ep cells (Fig. 2D and F). Together, these results indicate that starting with a lower number of Sf1Ep cells allows for greater overall yield and prolonged survival of T. pallidum subsp. *pallidum* per well, most likely because the lower initial Sf1Ep cell number delays the depletion of nutrients and the accumulation of toxic by-products.

### Effects of Sf1Ep cell concentration and passage number.

Prior studies had shown that substantial multiplication of T. pallidum in the *in vitro* culture system requires the presence of Sf1Ep cottontail rabbit epithelial cells, with only minimal increases (∼2-fold) observed in the absence of added Sf1Ep cells (22). A complication of these earlier studies was that small numbers of viable Sf1Ep cells were present in the inoculum from the prior culture, even if the inoculum was centrifuged twice at 100 × *g* to remove the mammalian cells. As a result, after 7 days of incubation, some of these “axenic” cultures contained Sf1Ep cells at <5% confluence, compared with >95% confluence in the standard cultures in which 1 × 10^5^ of the rabbit cells were added per well. To achieve truly axenic conditions, T. pallidum preparations that were frozen at 80°C in 15% glycerol (the agent used to preserve T. pallidum viability) were thawed and used for culture inoculation. This process renders Sf1Ep cells present in the inoculum nonviable because glycerol is not an effective cryopreservative for mammalian cells. We found that freezing the inoculum to destroy Sf1Ep cells results in no multiplication of T. pallidum and decreased motility. We now attribute the 2-fold multiplication observed previously (22) (when T. pallidum were passed directly without freezing) to the presence of residual Sf1Ep cells in the inoculum.

**FIG 3:**
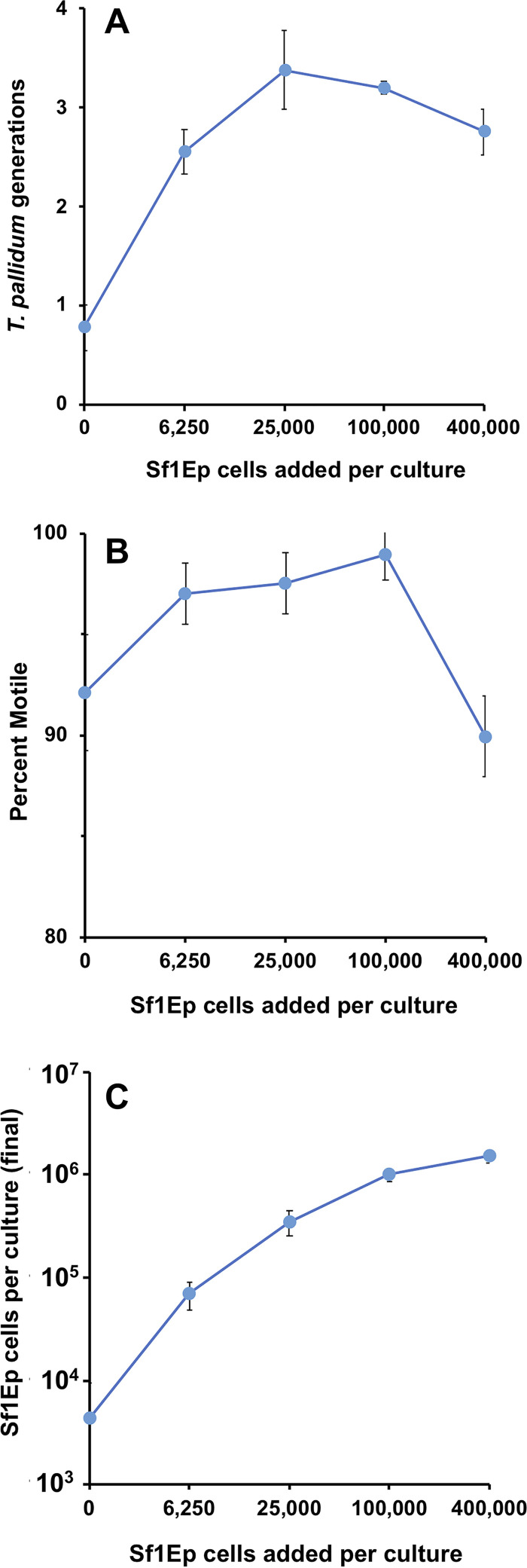
Relationship between the initial Sf1Ep cell numbers and multiplication of T. pallidum subsp. *pallidum* Nichols over a 7-day period of incubation. T. pallidum used for inoculating cultures were harvested from *in vitro* cultures, frozen at −80°C in 15% glycerol, and then thawed; this process effectively kills >99% of the Sf1Ep cells present in the inoculum. Triplicate cultures seeded with the number of Sf1Ep cells shown were inoculated with the same T. pallidum preparation (7.1 × 10^6^/culture) and harvested after 7 days of incubation. MOI ranged from 18 T. pallidum/cell at 400,000 Sf1Ep cells seeded to 1,131 T. pallidum/cell at 6,250 Sf1Ep cells seeded. (A) Cumulative number of T. pallidum generations (log_2_-fold increases). (B) Percent motile T. pallidum. (C) Number of Sf1Ep cells per culture at the time of harvest. All values represent the mean ± SEM for triplicate cultures.

To determine the effect of Sf1Ep cell number on T. pallidum growth and viability, we examined the multiplication and motility of T. pallidum in cultures containing a range of Sf1Ep cell concentrations. The results of a representative experiment using a frozen inoculum and varied initial Sf1Ep cell concentrations are shown in Fig. 3. In this case, triplicate cultures in 6-well plates were seeded with 0, 6.25 × 10^3^, 2.5 × 10^4^, 1 × 10^5^, or 4 × 10^5^ Sf1Ep cells/culture and a day later were inoculated with 250 μl of a T. pallidum subsp. *pallidum* Nichols frozen preparation; the cultures were then incubated 7 days under otherwise standard conditions. Multiplication of T. pallidum is shown as the number of generations (doublings) occurring during the incubation period. Optimal growth (Fig. 3A) and motility (Fig. 3B) were obtained at initial Sf1Ep cell concentrations of 2.5 × 10^4^ and 1 × 10^5^ cells per culture, with tapering of the yield (and, to a lesser extent, motility) at lower and higher Sf1Ep cell numbers. Cultures with no added Sf1Ep cells exhibited less than a 2-fold increase in T. pallidum numbers and relatively poor motility. Less multiplication of T. pallidum and lower motility were also seen at the highest concentration of Sf1Ep cells. The number of Sf1Ep cells recovered at the end of the incubation period was asymptotic at ∼1 × 10^6^ culture at the higher initial concentrations (Fig. 3C); confluence was approximately 0%, 20%, 60%, 95%, and 100%, respectively, in the cultures containing low to high Sf1Ep concentrations. Less than 10^4^ cells per culture were present in the trypsinized cultures from the axenic wells; these cells were most likely nonviable, in that no adherent cells were observed. It was surprising that 6.25 × 10^3^ cells were sufficient to obtain significant T. pallidum multiplication: these Sf1Ep cells were only 20% confluent in these cultures at the end of the experiment. T. pallidum is thus able to survive and multiply at a broad range of multiplicity of infection (MOI) values (18 to 1,131 T. pallidum per Sf1Ep cell) (Fig. 3). It is likely that the decreased yield of T. pallidum in the wells seeded with 4 × 10^5^ Sf1Ep cells was due to early confluence and rapid nutrient depletion in these cultures. Based on these and other studies, we routinely seed cultures with 5 × 10^4^ to 1 × 10^5^ Sf1Ep cells/well in standard 6-well plates (9.5 cm^2^/well), with proportionally higher or lower numbers based on the surface area of the culture well or flask.

T. pallidum has been shown to grow successfully in coculture with Sf1Ep cells between passages 19 to 50 (22). With successive passages, the Sf1Ep cultures grow more quickly and achieve higher densities, indicating selection of more rapidly dividing subpopulations. The higher passages of this secondary cell culture appear to have undergone spontaneous transformation, in that they apparently can be passaged indefinitely. To determine if Sf1Ep cell passage number influences T. pallidum growth and motility *in vitro*, T. pallidum Nichols was cocultured in parallel with passage 20 (P20) and P50 Sf1Ep cells. There was no difference in T. pallidum fold increase or motility in the P20 and P50 cultures after 21 days of growth and three passages (see Fig. S1 in the supplemental material). Some studies indicated that cultures with very high passage number Sf1Ep cells (e.g., P71) result in lower yields of T. pallidum (data not shown). Therefore, we recommend that low passage number stocks of Sf1Ep cells be maintained by freezing in liquid nitrogen and that the rabbit epithelial cell cultures only be used up to passage 60.

10.1128/mBio.03536-20.1 FIG S1 Growth of T. pallidum subsp. *pallidum* Nichols is nearly equivalent in Sf1Ep cell cultures of passage number 20 (P20) and P50. The same preparation of T. pallidum was inoculated and subcultured in triplicate wells containing TpCM-2 and either P20 or P50 cells. (A) Increases in T. pallidum per culture over three *in vitro* passages. (B) Cumulative generations of T. pallidum occurring over the 21-day period of incubation. Percent motility was consistently ≥99% over the course of the experiment. Download FIG S1, PDF file, 0.1 MB. Copyright © 2021 Edmondson et al. 2021 Edmondson et al. https://creativecommons.org/licenses/by/4.0/ This content is distributed under the terms of the Creative Commons Attribution 4.0 International license.

### Long-term culture requires nutrients deficient in Eagle’s MEM.

We had hypothesized previously that the recent success in long-term cultivation of T. pallidum was due the replacement of Eagle’s MEM in the prior T. pallidum culture medium (TpCM-1) with the more complex CMRL 1066 medium in TpCM-2 (22). To examine this hypothesis, we directly compared the survival and multiplication of T. pallidum subsp. *pallidum* Nichols in TpCM-1 and TpCM-2 under otherwise identical conditions. In addition, another complex tissue culture medium, M199, was included; the resulting medium was called TpCM-3. These three media are identical except for the differences in basal media.

**FIG 4:**
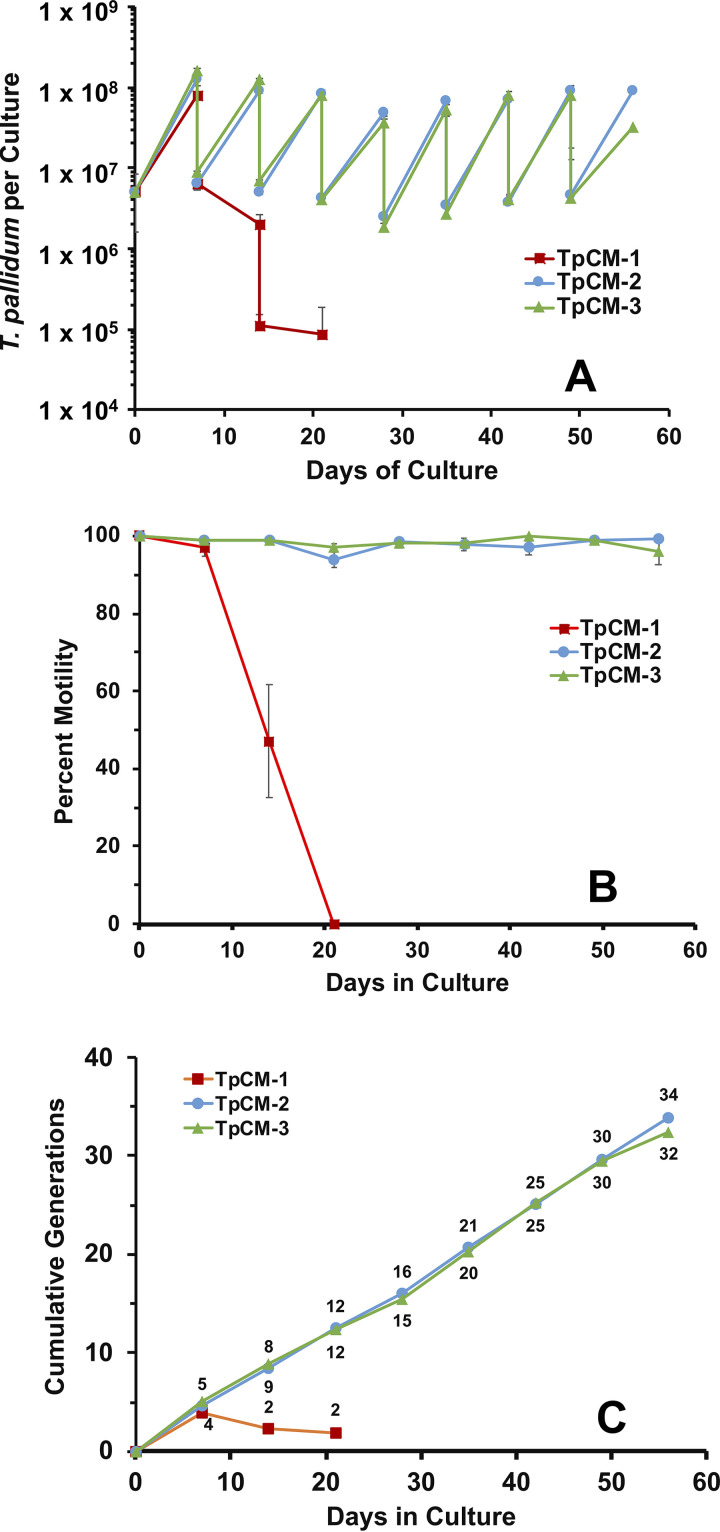
Long-term survival and multiplication of T. pallidum subsp. *pallidum* Nichols is dependent on the nutrient composition of the basal medium. In this study, the same preparation of spirochetes (from TpCM-2 *in vitro* cultures) was passaged in parallel in T. pallidum culture media containing Eagle’s MEM (TpCM-1), CMRL 1066 (TpCM-2), and M199 (TpCM-3) as the basal medium and examined for up to 7 *in vitro* passages. Error bars represent SEM between triplicate cultures. (A) Sawtooth plot shows continued multiplication in TpCM-2 and TpCM-3 but the rapid decline of T. pallidum cultured in TpCM-1 after the first passage. (B) Percent motile T. pallidum in the cultures. (C) Cumulative number of T. pallidum generations in the three media.

The results from one such study are shown in Fig. 4. As expected, T. pallidum subsp. *pallidum* Nichols cultured in TpCM-2 continued to multiply throughout the 56-day course of this experiment, retained >96% motility throughout, and underwent an average of 34 cell divisions (average generation time of 39.6 h). In contrast, treponemes cultured in TpCM-1 multiplied well during the first 7-day culture period but declined rapidly thereafter in terms of both T. pallidum per culture and motility. Two additional experiments comparing growth of T. pallidum subsp. *pallidum* Nichols in TpCM-1 and TpCM-2 over a 21-day period yielded the same pattern, although in one of these experiments, multiplication extended into the second week of culture (data not shown). The M199-based medium TpCM-3 yielded comparable results to TpCM-2 (Fig. 4), with very similar growth patterns, retention of motility, and average generation time (41.4 h). These results demonstrate that nutrients present in CMRL 1066 and M199 media but lacking in Eagle’s MEM are required for long-term survival and growth of T. pallidum. This finding was somewhat surprising, given that the additional ingredients present in CMRL 1066 and M199 media differ significantly from one another (see Discussion).

This analysis was expanded to the T. pallidum subsp. *pallidum* strains SS14 and UW231B (see Fig. S2 in the supplemental material). In addition, we examined whether a T. pallidum culture medium containing a 50:50 combination of CMRL 1066 and M199 basal media (called TpCM-4) resulted in enhanced growth. This approach was based on the hypothesis that the different ingredients present in these basal media may have an additive effect on T. pallidum survival and multiplication. The Nichols, SS14, and UW231B strains each exhibited quite similar growth patterns in TpCM-2, TpCM-3, and TpCM-4, although there was a trend toward decreased multiplication in later passages for the SS14 and UW231B strains in the M199-based medium TpCM-3 (Fig. S2). No growth of T. pallidum Nichols, SS14, or UW231B strains was observed in the absence of Sf1Ep cells in any of the three media (see Fig. S3 in the supplemental material).

10.1128/mBio.03536-20.2 FIG S2 Media based on CMRL 1066 (TpCM-2), M199 (TpCM-3), and equal amounts of CMRL 1066 and M199 (TpCM-4) support the survival and multiplication of T. pallidum subsp. *pallidum* strains SS14 and UW231B. Experiments were conducted in the same manner as described for Fig. 4. (A to C) Strain SS14. (D to F) Strain UW231B. T. pallidum per culture (A, D), percent motility (B, E), and cumulative generations (C, F) are shown. Download FIG S2, PDF file, 0.06 MB. Copyright © 2021 Edmondson et al. 2021 Edmondson et al. https://creativecommons.org/licenses/by/4.0/ This content is distributed under the terms of the Creative Commons Attribution 4.0 International license.

10.1128/mBio.03536-20.3 FIG S3 Lack of growth of T. pallidum subsp. *pallidum* strains Nichols, SS14, and UW231B under axenic conditions. Inocula of T. pallidum were harvested from *in vitro* cultures, frozen at –80°C in 15% glycerol, and then thawed to render any Sf1Ep cells present nonviable. The same preparation was used to inoculate parallel cultures containing TpCM-2 and Sf1Ep cells (as a positive control), or TpCM-2, TpCM-3, or TpCM4 without added Sf1Ep cells. Results represent the cumulative log_2_ change from the number of T. pallidum in the initial inoculum; a change of +1 represents one doubling of cell numbers. None of the axenic cultures exhibited a substantial increase in the number of T. pallidum. Download FIG S3, PDF file, 0.03 MB. Copyright © 2021 Edmondson et al. 2021 Edmondson et al. https://creativecommons.org/licenses/by/4.0/ This content is distributed under the terms of the Creative Commons Attribution 4.0 International license.

The effect of replacing CMRL 1066 with another complex tissue culture medium, MCDB, was also examined in the Sf1Ep coculture system. The MCDB-based medium did not support any multiplication of T. pallidum Nichols, and motility was reduced to 49% on day 7 compared with 99% for the TpCM-2 control (data not shown). However, the Sf1Ep cells survived and grew well in this medium.

We have begun the process of further defining the nutrients required for long-term T. pallidum multiplication. As a first step, we have examined some of components that are lacking in the nonsupportive Eagle’s MEM but present in the effective CMRL 1066. For example, several nucleic acid precursors present in CMRL 1066 along with additional nucleic acid precursors (see Table S2 in the supplemental material) were added in 1× and 2× concentrations to TpCM-1 and TpCM-2 to determine what effect this addition would have on T. pallidum survival and multiplication. The addition of these nucleic acid precursors did not improve the retention of motility or growth in TpCM-1, and supplementation of TpCM-2 with higher quantities of these compounds actually decreased multiplication of T. pallidum (see Fig. S4 in the supplemental material). Thus, supplementation with the nucleic acid precursors was not sufficient to support long-term multiplication in TpCM-1, and other (or additional) components present in TpCM-2 may be needed to provide the required nutrients.

10.1128/mBio.03536-20.4 FIG S4 Supplementation of TpCM-1 and TpCM-2 with a nucleic acid precursor mixture does not improve T. pallidum growth *in vitro*. A 1,000× solution of the nucleic acid precursor compounds present in CMRL 1066 but absent in Eagle’s MEM (Table S2) was added to TpCM-1 (A, B) and TpCM-2 (C, D) at either 1× or 2× concentration and compared with the same media without the additive; these media were utilized throughout the 28-day course of incubation. (A, C) Average number of T. pallidum per culture ± SE. (B, D) Average percent motility of T. pallidum from three samples. Download FIG S4, PDF file, 0.07 MB. Copyright © 2021 Edmondson et al. 2021 Edmondson et al. https://creativecommons.org/licenses/by/4.0/ This content is distributed under the terms of the Creative Commons Attribution 4.0 International license.

10.1128/mBio.03536-20.9 TABLE S2 Components present in the 1,000× nucleotide/nucleoside solution. Download Table S2, PDF file, 0.3 MB. Copyright © 2021 Edmondson et al. 2021 Edmondson et al. https://creativecommons.org/licenses/by/4.0/ This content is distributed under the terms of the Creative Commons Attribution 4.0 International license.

Fieldsteel et al. (20, 25) introduced the use of resazurin in T. pallidum culture media. This compound is a useful color indicator of both redox potential and pH, turning from blue to pink in the transition from air to 1.5% O_2_ and to yellow as the medium acidifies (usually after day 7 to 9). However, most available culture media contain phenol red as a pH indicator. To determine if phenol red has an effect on long-term *in vitro* culture in the presence of Sf1Ep cells, the multiplication and motility of T. pallidum Nichols in control TpCM-2 with resazurin was compared with that of organisms incubated in TpCM-2 containing phenol red. T. pallidum growth in the presence of phenol red was significantly reduced compared with the control at the first passage (7 days of culture) (see Fig. S5 in the supplemental material). Growth continued to decrease throughout the 21-day experiment. Although the growth of T. pallidum was inhibited by phenol red beginning in the first 7 days, motility was not significantly decreased until day 21 (74% motile versus 99% motile for the control).

10.1128/mBio.03536-20.5 FIG S5 Phenol red inhibits the *in vitro* growth of T. pallidum subsp. *pallidum* Nichols. (A) Comparison of T. pallidum growth between standard TpCM-2 medium containing resazurin and TpCM-2 containing phenol red. Average number of T. pallidum per culture from three replicate cultures is shown. (B) Average percent motility of T. pallidum per culture. Data represent the mean ± SE of three replicate cultures. Download FIG S5, PDF file, 0.02 MB. Copyright © 2021 Edmondson et al. 2021 Edmondson et al. https://creativecommons.org/licenses/by/4.0/ This content is distributed under the terms of the Creative Commons Attribution 4.0 International license.

Finally, we have found that TpCM-2 stored frozen at –20°C is as effective as freshly prepared TpCM-2 in supporting the survival and multiplication of T. pallidum Nichols. Excess freshly prepared TpCM-2 from other experiments was frozen following the overnight pre-equilibration in 1.5% O_2_, 5% CO_2_, and 93.5% N_2_. In one experiment, T. pallidum Nichols from an *in vitro* culture was used to inoculate triplicate cultures containing fresh TpCM-2 or two different lots of frozen TpCM-2; the cultures were then evaluated 7 days later. The results were nearly identical, with average yields of 1.16 × 10^8^, 1.33 × 10^8^, and 1.30 × 10^8^ (21.7- to 25.0-fold increase) with 99% motility for the fresh and two frozen media, respectively (data not shown). In a second experiment, a T. pallidum culture was inoculated into two parallel cultures maintained in fresh and frozen TpCM-2 for three successive passages. Growth over the 3-week period was nearly equivalent, with average generation times (with ± SE) being 38.9 ± 1.0 h and 40.6 ± 2.9 h (*P* = 0.55) in the fresh and frozen medium conditions, respectively, and motility being consistently ≥98% (data not shown). Thus, it is possible to simplify the T. pallidum culture procedure by using medium stored at −20°C.

### Serum requirement.

**FIG 5:**
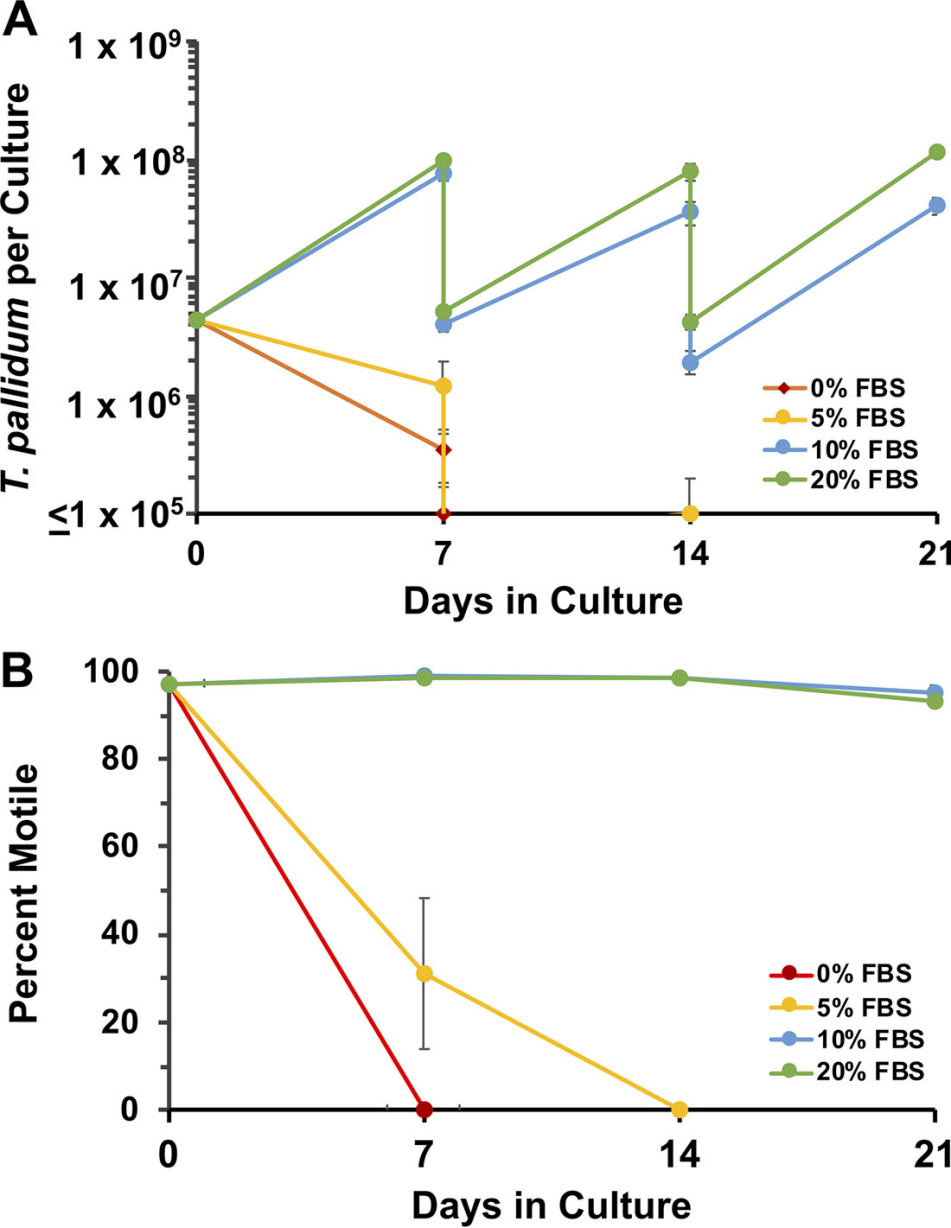
Requirement for fetal bovine serum (FBS) in TpCM-2 for support of T. pallidum subsp. *pallidum* Nichols long-term *in vitro* survival and multiplication. Triplicate parallel cultures at each serum concentration were monitored over three 7-day passages. (A) T. pallidum per culture, showing the culture yield and inoculum for the next passage at each time point. (B) Percent motility of T. pallidum. Values represent the mean ± SEM from three biological replicates.

The standard formulation of TpCM-2 contains 20% heat-inactivated fetal bovine serum (FBS), as did the original TpCM-1 formulation (26, 27). To determine if T. pallidum can be cultivated in TpCM-2 containing less FBS, T. pallidum was inoculated into TpCM-2 containing 0%, 5%, 10%, and 20% FBS. Within 7 days, the T. pallidum cultures in TpCM-2 without FBS had decreased in numbers and were entirely nonmotile (Fig. 5). TpCM-2 containing 5% FBS was also unable to support long-term T. pallidum survival and growth, with organisms becoming nonmotile after 14 days. Multiplication of T. pallidum in TpCM-2 containing 10% FBS was reduced somewhat compared with standard TpCM-2 (20% FBS), but growth was sustained throughout the course of the experiment and the organisms remained highly motile (95%). Thus, 20% FBS was the optimal concentration of those tested.

### Atmospheric conditions.

**FIG 6:**
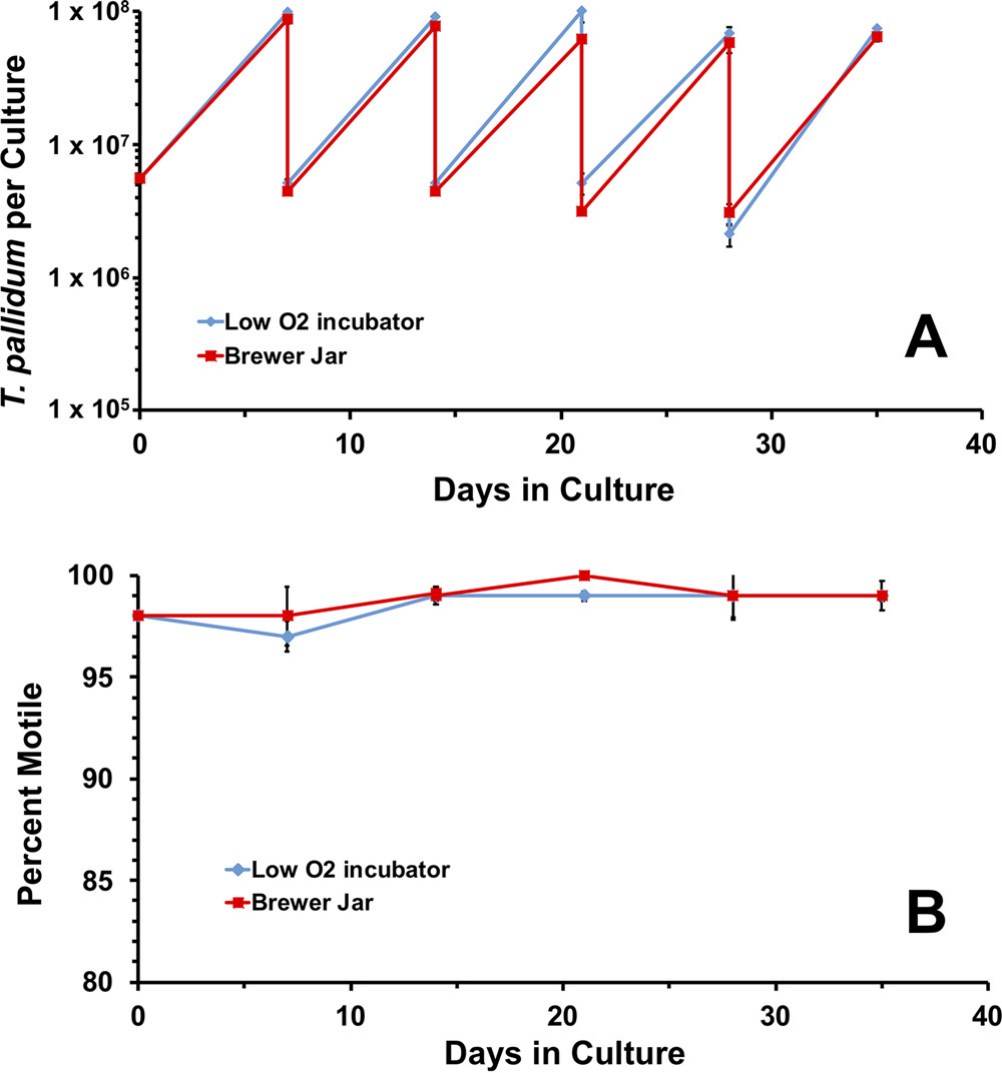
T. pallidum subsp. *pallidum* Nichols can be cultured effectively long-term *in vitro* in Brewer jars. A T. pallidum preparation was used to inoculate duplicate 6-well plates containing Sf1Ep cells and TpCM-2 medium. The cultures were incubated in parallel in a Brewer jar equilibrated with 1.5% O_2_: 5% CO_2_: balance N_2_ or a tri-gas incubator containing the same gas mixture at 34°C and were passaged in the same manner at 7-day intervals. Results represent the average T. pallidum per culture and percent motility ± SEM for triplicate cultures in each environment.

T. pallidum cultures have been maintained routinely in a low-oxygen tri-gas (N_2_:O_2_:CO_2_) incubator (22). As an alternative to this specialized incubator, cultures were incubated in Brewer jars evacuated and refilled with 1.5% O_2_, 5% CO_2_, and 93.5% N_2_ as described in the Materials and Methods. The Brewer jars were placed in a standard incubator set at 34°C, and T. pallidum multiplication and motility were compared with those of parallel cultures incubated in a tri-gas incubator. Over the course of 35 days, the number of T. pallidum per culture and motility were highly comparable between parallel cultures incubated in the Brewer jar or a tri-gas incubator (Fig. 6). Over the course of this experiment, there were 21.5 cumulative generations in the low-oxygen incubator and 20.5 in the Brewer jar, corresponding to similar generation times of 39.1 h and 40.9 h, respectively. These results indicate that T. pallidum can be successfully cultured in a Brewer jar as well as in the specialized incubator.

Cox et al. (28) had shown previously that T. pallidum growth in short-term *in vitro* cultures was optimal in the range of 1% to 5% oxygen. To determine if increasing the percent oxygen in the environment has an effect on long-term T. pallidum growth, cultures grown in TpCM-2 were exposed to either 1.5% or 3% oxygen, using parallel cultures in two different tri-gas incubators. Both cultures were successfully maintained for 42 days, with no decrease in T. pallidum growth or motility at the higher oxygen concentration (see Fig. S6 in the supplemental material).

10.1128/mBio.03536-20.6 FIG S6 Survival and growth of T. pallidum subsp. *pallidum* Nichols are highly similar in the presence of 1.5% O_2_ or 3.0% O_2_. Triplicate cultures were incubated and subcultured under standard conditions using TpCM-2 in the Sf1Ep coculture system, except that the cultures were maintained in parallel in two tri-gas incubators containing either 1.5% O_2_:5% CO_2_:balance N_2_ or 3% O_2_:5% CO_2_:balance N_2_. (A) T. pallidum per culture over the 28-day course of incubation and subculture. (B) Percent motility. (C) Cumulative increase in T. pallidum generations. Download FIG S6, PDF file, 0.05 MB. Copyright © 2021 Edmondson et al. 2021 Edmondson et al. https://creativecommons.org/licenses/by/4.0/ This content is distributed under the terms of the Creative Commons Attribution 4.0 International license.

### Oxygen radical-scavenging reagents are not essential for T. pallidum survival and growth in the presence of Sf1Ep cells.

Dithiothreitol (DTT) and other reducing agents that neutralize reactive oxygen species (ROS) have long been known to extend the survival of T. pallidum in *in vitro* systems that lack mammalian cells (19, 20, 29–34). Therefore, DTT was utilized in the initial culture medium described by Fieldsteel et al. (20). Cox (19) also included high concentrations of the oxygen-scavenging reagents d-mannitol (0.88 mM) and l-histidine (0.52 mM) in the medium TpCM-1, based on ∼20% increases in T. pallidum yields in Sf1Ep cell cocultures. We carried out a series of experiments to determine whether these three reagents are necessary to obtain optimal long-term survival and multiplication of T. pallidum Nichols.

**FIG 7:**
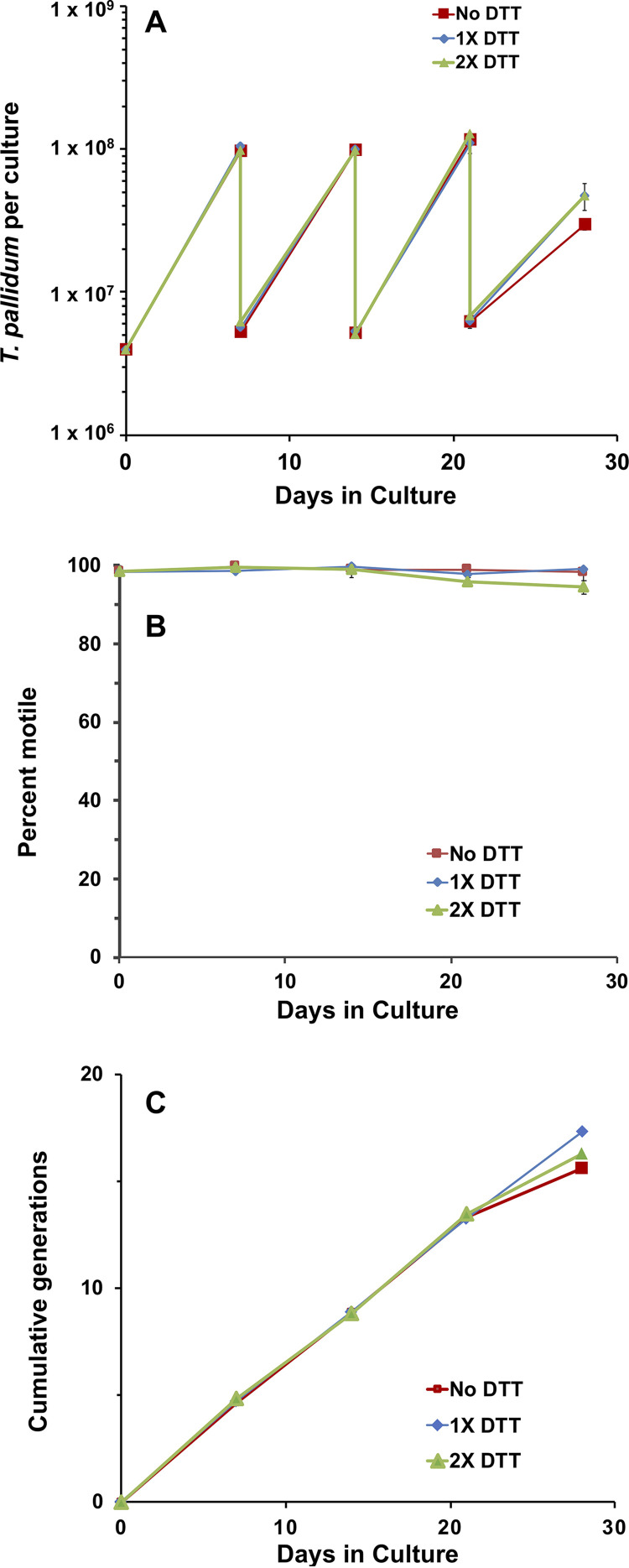
The absence of dithiothreitol (DTT) does not dramatically affect the multiplication or motility of T. pallidum under standard passage conditions. T. pallidum subsp. *pallidum* Nichols freshly harvested from *in vitro* cultures was transferred to new cultures with Sf1Ep cells and TpCM-2 containing either no DTT, the usual concentration of DTT (0.52 mM, 1× DTT), or double the concentration of DTT (1.04 mM, 2× DTT). T. pallidum concentration (A) and motility (B) were monitored over a total of four passages in which the no DTT, 1× DTT, and 2× DTT conditions were maintained. (C) Cumulative number of T. pallidum generations. Results represent the mean of three replicate cultures under each condition (± SEM for T. pallidum per culture and percent motile values).

TpCM-2 contains 0.52 mM DTT (22). To determine if DTT is important for long-term *in vitro* culture of T. pallidum, cultures containing no DTT, 1× DTT (0.52 mM), and 2× DTT (1.04 mM) were assessed for growth and motility over 28 days (Fig. 7). The growth patterns and motility under these three conditions were virtually superimposable over the first 21 days. At 28 days, the growth of T. pallidum in TpCM-2 containing no DTT was reduced somewhat compared with that for organisms grown in standard TpCM-2 for unknown reasons.

**FIG 8:**
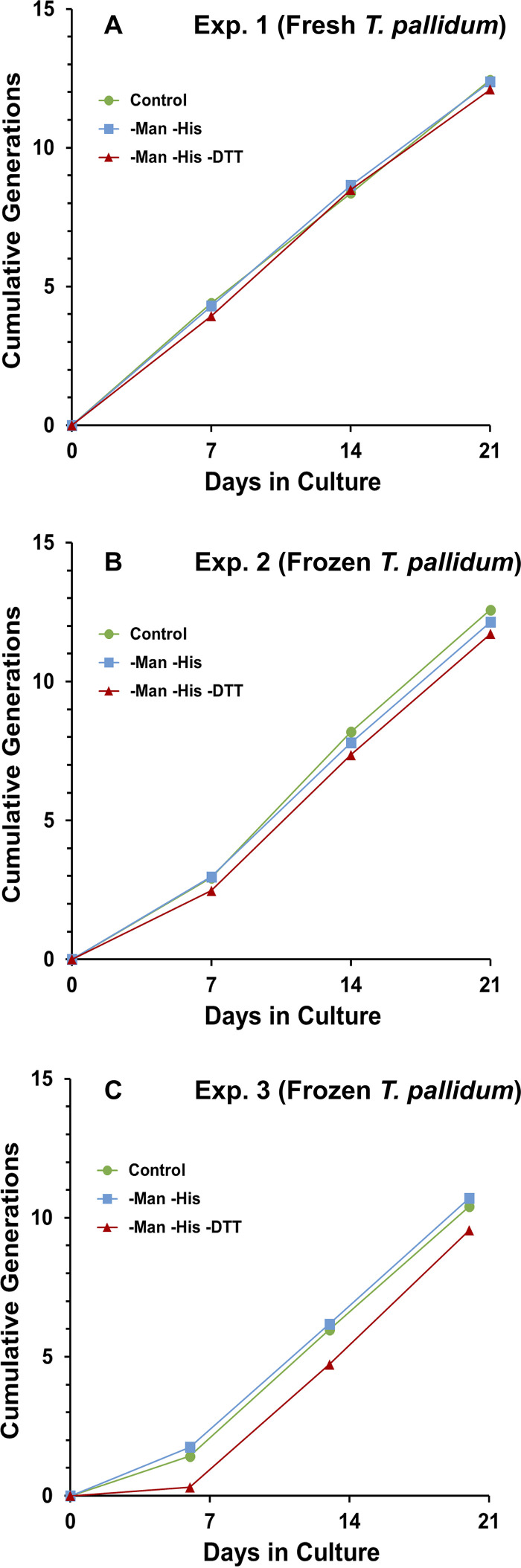
The presence of the reactive oxygen species (ROS)-scavenging medium components dithiothreitol (DTT), d-mannitol (Man), and l-histidine (His) is not required for *in vitro* survival and multiplication of T. pallidum subsp. *pallidum* Nichols. Cultures were inoculated with T. pallidum either freshly harvested from *in vitro* cultures (A, experiment 1) or frozen and then thawed immediately prior to inoculation (B and C, experiments 2 and 3). Parallel cultures contained Sf1Ep cells and either TpCM-2 (control) or the same medium preparation lacking Man and His (−Man −His) or Man, His, and DTT (−Man −His −DTT). The average numbers of cumulative generations for triplicate cultures under each condition are shown. Note that the first passage of cultures in experiment 3 was performed at 6 days rather than the standard 7 days.

It was possible that DTT is not required for T. pallidum survival and multiplication due to the presence of d-mannitol and l-histidine in TpCM-2. To test this hypothesis, we conducted a series of three experiments comparing the control TpCM-2 (with the three ROS-scavenging components) to the same medium lacking d-mannitol and l-histidine (−Man −His) or lacking all three compounds (−Man −His −DTT). In this series, experiment 1 used freshly harvested *in vitro* cultured T. pallidum subsp. *pallidum* Nichols as the inoculum, whereas experiments 2 and 3 utilized cultured Nichols organisms that had been frozen at –80°C in the presence of 15% glycerol and thawed just prior to inoculation. Comparable growth rates (shown as cumulative generations) were obtained with all three media after the first 7-day period (Fig. 8). However, it is of interest that growth of the frozen stocks of T. pallidum was reduced during the initial 7-day culture period in the cultures lacking DTT. This result suggests that the presence of DTT in some way counteracts the stress introduced by the freeze-thaw process. In experiments 2 and 3, additional parallel cultures were incubated in the absence of Sf1Ep cells in the same three media. These axenic cultures did not exhibit any increase in T. pallidum numbers above the inoculum and were greatly reduced in both concentration and motility by day 14 of incubation, regardless of whether DTT, d-mannitol, or l-histidine were present (data not shown).

### Antimicrobial agents.

The standard T. pallidum culture system does not contain antimicrobial agents, and fungal or bacterial contamination of the cultures can occur. For this reason, we determined if antimicrobial agents could be utilized in the long-term T. pallidum culture system to prevent contamination with other organisms without negatively impacting T. pallidum growth or motility. First, an antimicrobial agent “cocktail” (final concentrations of 2.5 μg/ml amphotericin B, 50 μg/ml rifampin, and 20 μg/ml phosphomycin) commonly used in the cultivation of the spirochete Borrelia burgdorferi from animal specimens was added to TpCM-2. T. pallidum Nichols growth and motility were significantly decreased upon the addition of this antimicrobial solution, with the cultures dying out by 21 days (see Fig. S7A and B in the supplemental material).

10.1128/mBio.03536-20.7 FIG S7 Multiplication of T. pallidum subsp. *pallidum* Nichols is inhibited by an antibiotic mixture utilized to prevent contamination of *Borrelia* cultures. (A, B) Addition of a “cocktail” of 2.5 μg/ml amphotericin B, 50 μg/ml rifampicin, and 20 μg/ml phosphomycin resulted in reduced multiplication and retention of motility of T. pallidum during incubation in TpCM-2 in the Sf1Ep coculture system. (C, D) Amphotericin B (2.5 μg/ml) alone does not interfere with T. pallidum growth or motility. Multiplication continued after removal of amphotericin B (amphotericin B to TpCM2). Results are the mean ± SE from triplicate cultures under each condition. Subcultures were performed at 7-day intervals with the transfer of a constant volume of 250 μl from each culture. Download FIG S7, PDF file, 0.07 MB. Copyright © 2021 Edmondson et al. 2021 Edmondson et al. https://creativecommons.org/licenses/by/4.0/ This content is distributed under the terms of the Creative Commons Attribution 4.0 International license.

We next determined whether individual antimicrobial agents could be added to TpCM-2 without affecting T. pallidum viability or multiplication. The antifungal agent amphotericin B at a final concentration of 2.5 μg/ml in TpCM-2 did not decrease growth or motility (Fig. S7C and D), and T. pallidum was maintained continuously for 70 days. To determine if T. pallidum cultures could be treated with amphotericin B and then transitioned back into unsupplemented TpCM-2 (mimicking the treatment of a contaminated culture), TpCM-2 without antibiotics was inoculated with T. pallidum from the amphotericin B-treated culture. Cultures treated with amphotericin B were successfully transitioned back into unsupplemented TpCM-2 with no loss in growth or motility (Fig. S7C and D).

**FIG 9:**
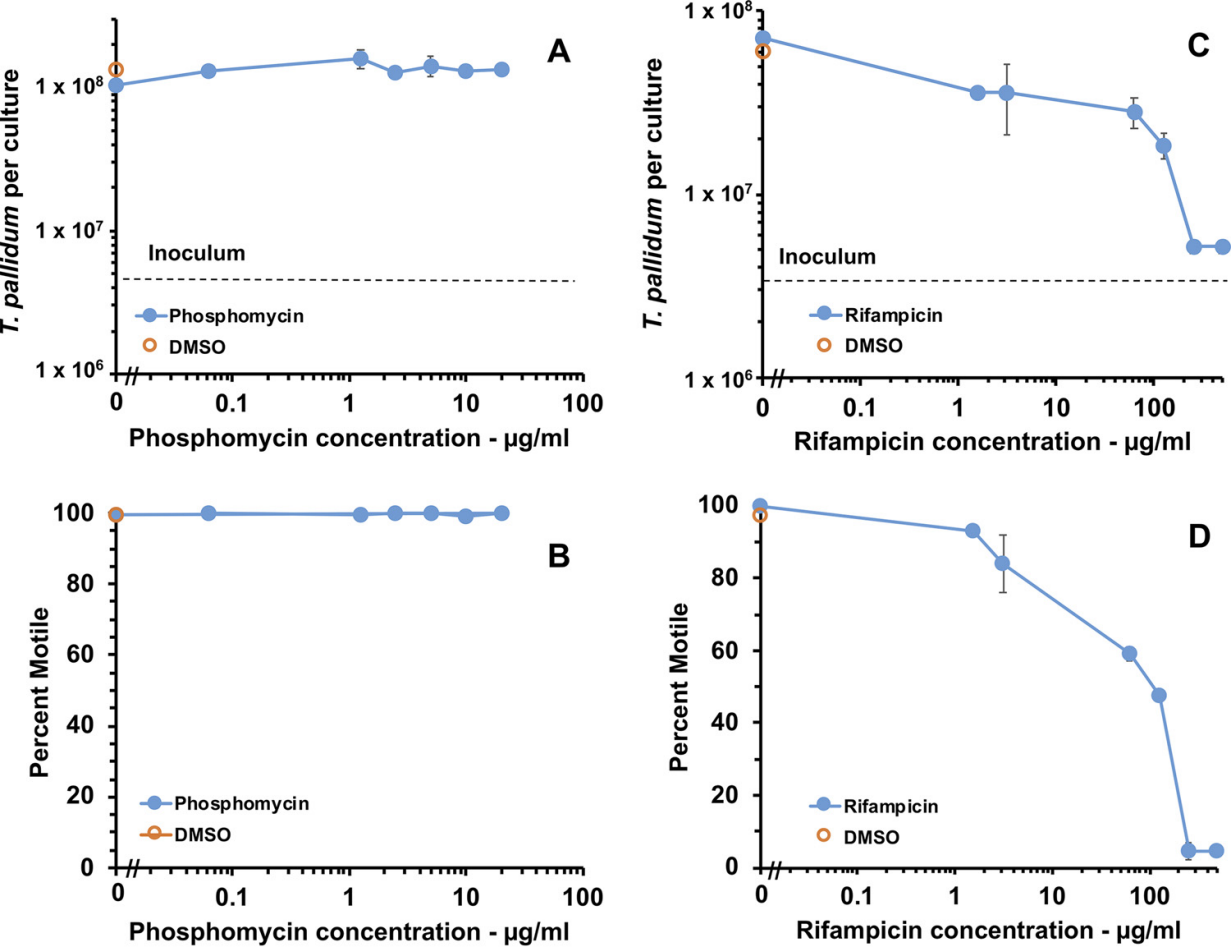
T. pallidum growth *in vitro* is reduced upon addition of rifampicin but not phosphomycin. Triplicate cultures at each antibiotic concentration were inoculated with T. pallidum subsp. *pallidum* Nichols and incubated for 7 days in the Sf1Ep culture system with TpCM-2 under standard conditions. The effects of phosphomycin (A, B) and rifampicin (C, D) were examined in two separate experiments. (A, C) Average number of T. pallidum per culture ± SEM from three samples. Dashed line indicates the inoculum level. (B, D). Average percent motility of T. pallidum ± SEM from three samples.

**FIG 10:**
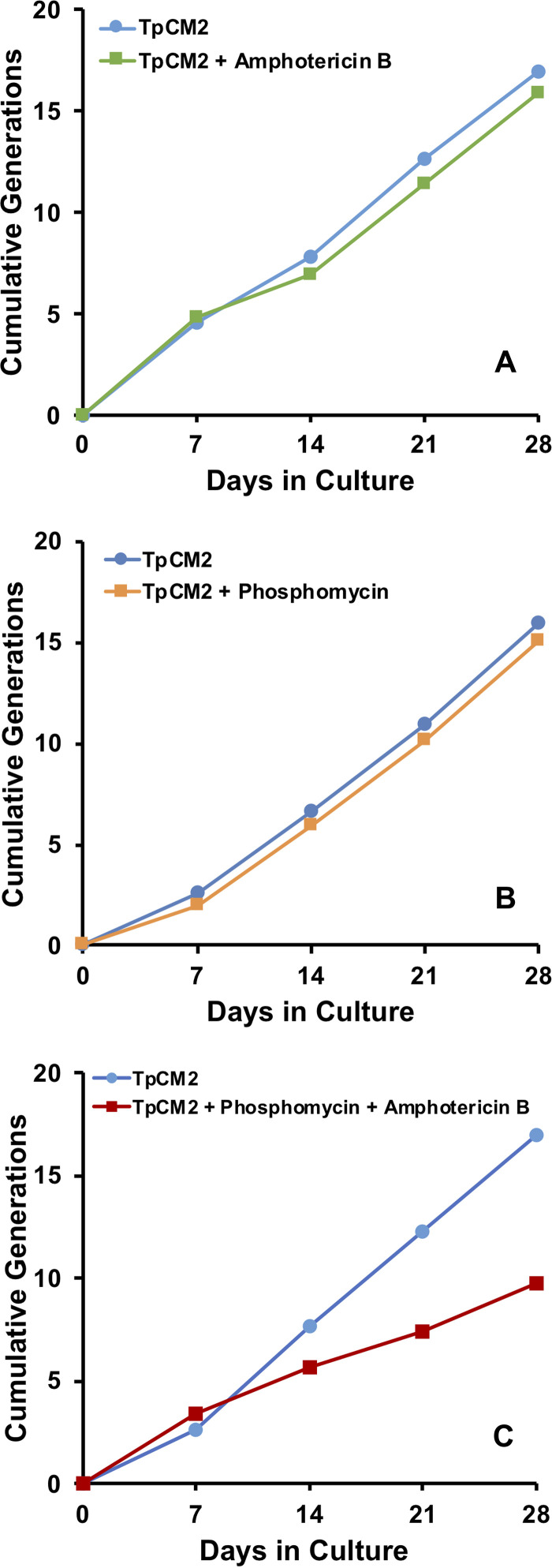
Effects of the presence of amphotericin B and phosphomycin on the long-term culture of T. pallidum subsp. *pallidum* Nichols. Subculture was performed at 7-day intervals in the constant presence or absence of 2.5 μg/ml amphotericin B (A), 20 μg/ml phosphomycin (B), or both 2.5 μg/ml amphotericin B and 20 μg/ml phosphomycin (C). Data are shown as the cumulative number of generations over the 28-d period of the experiments.

These results indicated that either rifampicin or phosphomycin were detrimental to T. pallidum survival or multiplication. To determine the inhibitory concentrations of these antibiotics, T. pallidum Nichols was exposed to a range of concentrations of rifampicin and phosphomycin. Addition of phosphomycin at concentrations ranging from 0.0625 μg/ml to 20 μg/ml did not decrease T. pallidum growth or motility compared with the DMSO (drug vehicle) and TpCM-2 controls (Fig. 9A and B). In contrast, rifampicin reduced T. pallidum growth and motility compared with the DMSO and TpCM-2 controls at all concentrations studied (1.56 to 500 μg/ml) (Fig. 9C and D). Phosphomycin (20 μg/ml) and amphotericin B (2.5 μg/ml) were not inhibitory to T. pallidum growth by themselves, as indicated by the cumulative generation curves shown in Fig. 10A and B. However, the combination of these two antibiotics impeded T. pallidum growth and motility (Fig. 10C), suggesting that when provided together, they synergistically inhibit T. pallidum viability. Therefore, only one of these antimicrobial agents should be added to the TpCM-2 culture system at a time based upon which contaminating microbes are a greater problem in the culture system, namely, bacteria or fungi. Alternatively, concentrations could be adjusted to levels that do not affect T. pallidum but still inhibit growth of other microbes.

## DISCUSSION

In this study, we report that T. pallidum subsp. *pallidum* Nichols has now been cultured continuously *in vitro* for over 3 years, indicating that this organism can be propagated indefinitely in the Sf1Ep coculture system. Several additional strains, including the syphilis isolates SS-14, UW231B, UW249B, and Mexico A and the bejel strain Bosnia A, have been cultured *in vitro* for over 200 days (Table 1). These results provide evidence that T. pallidum subsp. *pallidum* strains in both the Nichols and SS14 genetic clusters (35, 36) as well as T. pallidum subsp. *endemicum* strains can be maintained in this system. In addition, we found that the Nichols strain has shorter generation times than strains of the SS14 cluster and T. pallidum subsp. *endemicum* Bosnia A. Interestingly, in our hands, the Nichols strain also reaches peak infection in rabbits sooner than the other strains. The Nichols strain has been extensively passaged in rabbits over many years and likely adapted for rapid growth, perhaps “conditioning” it for adaptation to coculture with rabbit cells.

Yaws isolates have not as yet been cultured successfully *in vitro*. Only a few attempts have been made to propagate T. pallidum subsp. *pertenue* strains Gauthier and Samoa F by this means, so it is unclear whether the negative results obtained thus far are due to technical issues or biologic differences. In prior studies, Cox and colleagues (20, 37) obtained survival and multiplication for periods up to 12 days of the T. pallidum subsp. *pallidum* KKJ, T. pallidum subsp. *pallidum* Mexico A, and T. pallidum subsp. *endemicum* Bosnia A. However, they observed only a limited increase (1.7- to 1.9-fold) in numbers of the T. pallidum subsp. *pertenue* strains Gauthier and Fribourg-Blanc in short-term cultures (20, 37), consistent with our results. If significant physiologic differences exist in yaws isolates, it may be necessary to further refine the culture medium or incubation conditions to promote *in vitro* multiplication of this subspecies. Genomic comparisons of strains of the three subspecies reveal that a few genes (including those encoding a CDP-alcohol phosphatidyltransferase, a methyl-accepting chemotaxis protein, and the membrane-associated protein TprL) are identical among T. pallidum subsp. *pallidum* and T. pallidum subsp. *endemicum* strains and yet differ in T. pallidum subsp. *pertenue* strains (9). This information could provide clues regarding the genetic differences that may result in different *in vitro* growth characteristics.

We also sought to further define the requirements for long-term culture of T. pallidum in an effort to improve growth, simplify the medium and culture conditions, and explore alternative culture methods, such as the use of Brewer anaerobic jars and the inclusion of antibiotics to inhibit bacterial or fungal contamination. Our main findings are that (1) the presence of Sf1Ep cells is required to support T. pallidum growth, but high Sf1Ep cell numbers or passage number decrease survival and yield; (2) consistent culture of T. pallidum beyond the first passage requires nutrients lacking in Eagle’s MEM but present in the more complex basal media CMRL 1066 and M199; (3) fetal bovine serum is also required, and its optimal concentration is ≥20%; (4) O_2_ concentrations of 1.5% or 3% yield equivalent T. pallidum survival and growth, and Brewer jars can successfully replace the more complex tri-gas incubators for retaining permissive atmospheric conditions; (5) addition of the ROS scavengers DTT, d-mannitol, and l-histidine is not required for long-term culture of T. pallidum in this system; (6) inclusion of antibiotics to reduce fungal or bacterial contamination is possible without reducing T. pallidum survival or multiplication; and (7) frozen TpCM-2 may be substituted for fresh TpCM-2 if the medium is thoroughly equilibrated with the low O_2_ gas mixture. Sometimes, different culture conditions were not significantly different at the first passage, so we routinely tested each condition for at least three passages. This phenomenon is likely due to the slow generation time of T. pallidum. Thus far, axenic conditions that support long-term survival and multiplication of T. pallidum have not been identified.

Long-term *in vitro* growth of T. pallidum requires Sf1Ep cottontail rabbit epithelial cell coculture. The original Sf1Ep cells were derived from the ear of a cottontail rabbit and represent a slow-growing secondary cell culture; the cells typically stop dividing after ∼27 to 30 *in vitro* passages. Continuous passage of Sf1Ep cells in our laboratory has resulted in the derivation of Sf1Ep variants that appear to have become immortalized. In comparison to Sf1Ep secondary cell cultures, this cell line divides more rapidly and seemingly can be passaged indefinitely. In our study, T. pallidum could be cocultured with either low-passage, untransformed Sf1Ep cells or the more rapidly growing, higher passage Sf1Ep cells. Very high passages of the transformed Sf1Ep cells resulted in a decrease in T. pallidum yield and motility; we recommend use of Sf1Ep cells that have undergone fewer than a total of 60 culture passages.

Sf1Ep cells metabolize TpCM-2 medium components and start to acidify the medium after 8 to 9 d of culture, as indicated by a change of medium color. Therefore, we examined whether starting the culture with a lower number of Sf1Ep cells per well would increase T. pallidum yield or allow the culture to be passed at longer time intervals. Growth curves indicate that T. pallidum growth steadily increases to about day 8 of culture before leveling off and dropping after day 10. This decrease in culture yield after day 8 corresponds to acidification of the culture, as indicated by a change in the medium color. Starting the culture with more Sf1Ep cells per well resulted in lower culture yield and a faster decrease in motility, while lower numbers of Sf1Ep cells resulted in higher yields and preservation of motility after longer periods of culture. Together, these data indicate that the standard 7-day period between passing the culture can be extended up to 10 days if the starting number of Sf1Ep cells is low. Extension of the time between passages allows for some flexibility in the scheduling of experiments, and also may be useful in the initial isolation of T. pallidum strains from patients and other procedures that require longer growth periods.

We have confirmed our earlier hypothesis (22) that the additional nutrients present in CMRL 1066 but lacking in Eagle’s MEM were required to support long-term culture of T. pallidum. It was also found that another complex tissue culture medium, M199, was equivalent to CMRL 1066 in supporting the survival and growth of T. pallidum. A comparison of the medium components revealed that there are 23 additional compounds in CMRL 1066 that are absent in Eagle’s MEM; similarly, M199 contains 24 compounds not present in Eagle’s MEM, and many of them are different from the ingredients of CMRL 1066 (see Table S3 in the supplemental material). CMRL and M199 contain nucleotide precursors, vitamins, and two amino acids not present in Eagle’s MEM. Surprisingly, CMRL 1066 and M199 differ entirely in the composition of nucleotide precursors and in the presence or absence of several vitamins/cofactors (Table S3) and yet are both able to support T. pallidum growth. It is likely that multiple components in the CMRL 1066 and M199 media are necessary, i.e., that no single component is sufficient. Therefore, identification of these additional required components will be a complex task. To address this question, we have initiated a three-pronged approach as follows: a comparison of effective and noneffective medium compositions, construction of effective media with deletion of individual or multiple compounds, and metabolomic analysis of consumed and secreted products. As an example, Eagle’s MEM does not contain any nucleic acid precursors, and T. pallidum lacks genes encoding the enzymes needed for the *de novo* synthesis of nucleic acid bases. We supplemented Eagle’s MEM with a mixture of nucleic acid precursors present in the effective media (Table S2), but this supplementation did not promote long-term T. pallidum growth (Fig. S4). Thus, the compounds are not sufficient to supplement the deficiencies in the Eagle’s MEM-based medium, although they still may be a subset of the compounds required for continuous culture.

10.1128/mBio.03536-20.10 TABLE S3 Components present in Eagle’s MEM, and additional components present in CMRL 1066 and M199. Download Table S3, PDF file, 0.4 MB. Copyright © 2021 Edmondson et al. 2021 Edmondson et al. https://creativecommons.org/licenses/by/4.0/ This content is distributed under the terms of the Creative Commons Attribution 4.0 International license.

We confirmed prior studies (26, 27) indicating that FBS is required for T. pallidum survival and growth in the Sf1Ep coculture system and that 20% is the optimal serum concentration. Serum has also been shown to be required for many other mammalian host-dependent *Treponema* species (7). Evidence to date indicates that the required component of serum is the protein fraction and that this dependence may be due to the ability of serum albumin to serve as a detoxifying carrier of lipids and thiamine pyrophosphate (27, 38, 39). Experiments have been initiated to further define the role of serum in supporting the long-term culture of T. pallidum.

T. pallidum is a microaerophilic organism (28–30, 40–42) and must be cultivated under atmospheric oxygen concentrations in the range of 1% to 5% (19, 28). This environment is typically provided by maintaining the cultures in a specialized tri-gas incubator. We found that cultures were successfully maintained in Brewer jars filled with 1.5% O_2_, 5% CO_2_, and 93.5% N_2_ placed in a standard incubator with no decrease in culture yield or motility, allowing for the use of a standard incubator instead of a more complex tri-gas incubator. Likewise, we found no difference in T. pallidum growth between organisms grown at 1.5% or 3% oxygen, indicating some flexibility in this parameter. The biochemical pathways involving oxygen have not been elucidated but may involve O_2_ as an electron acceptor in the conversion of NADH to NAD^+^ by NADH oxidase as a means of maintaining redox balance in the cytoplasm (3).

T. pallidum is sensitive to damage due to reactive oxygen species and will rapidly become nonmotile and noninfectious if incubated axenically in the presence of oxygen; in such experiments, the presence of ROS scavengers, such as DTT, extends survival (28–30, 41). In the first successful short-term culture studies, Fieldsteel et al. (20) included DTT in the Sf1Ep cocultures; later, Cox (19) added d-mannitol and l-histidine as additional ROS scavengers to the medium TpCM-1. Catalase and superoxide dismutase were also included in this medium, but in our hands, these additives did not affect T. pallidum survival or multiplication; they therefore were not used in our medium. In the studies reported here, we were surprised to find that the DTT, d-mannitol, and l-histidine supplements could be omitted from TpCM-2 with no apparent reduction in long-term T. pallidum survival or growth (Fig. 7 and 8). In some experiments, growth was initially reduced in the absence of DTT when frozen preparations were used to inoculate the *in vitro* cultures (Fig. 8); thus, continued use of DTT in TpCM-2 is recommended, but it appears d-mannitol and additional l-histidine (beyond that present in the CMRL 1066 basal medium) can be omitted. The medium without these two additives has been called TpCM-2B to indicate this minor modification. We hypothesize that one of the activities provided by the Sf1Ep cells is scavenging ROS, thus protecting T. pallidum against their toxic effects.

One potential application of long-term culture is the propagation of T. pallidum strains directly from patient specimens; it is anticipated that many of these inocula (e.g., those from skin lesions) will contain other microbes that could result in culture contamination. Therefore, we examined whether an antimicrobial mixture used for B. burgdorferi culture consisting of amphotericin B, phosphomycin, and rifampicin can be added to T. pallidum cultures (11). The antimicrobial solution resulted in loss of the culture after 21 days, as did a combination of amphotericin B and phosphomycin or rifampicin (Fig. 10; Fig. S7). Amphotericin B or phosphomycin, when used alone, allowed for maintenance of T. pallidum (Fig. 9; Fig. S7). Therefore, one of these antimicrobial agents may be added to the culture at a time to combat either fungal (amphotericin B) or bacterial contamination (phosphomycin) as needed.

The basic parameters required for long-term culture of T. pallidum strains (such as the requirements for Sf1Ep cells, serum, and microaerobic levels of oxygen) are in agreement with the prior findings of Fieldsteel and coworkers (19, 20), confirming that their careful studies provided a firm foundation for the continuous culture of these fastidious organisms and further study of their complex physiologic requirements. Delineation of the required compounds in media supporting continuous culture of T. pallidum growth would further define the growth requirements of this organism. Approaches such as metabolomics may indicate the compounds or activities that Sf1Ep cells are providing, potentially leading to the ultimate development of axenic culture conditions. Overall, the T. pallidum culture system has been shown to be highly reproducible and robust, and our group and other laboratories have begun to apply this system to other aspects of T. pallidum biology, including antimicrobial susceptibility (43), isolation of clonal populations, propagation of organisms from patient samples, and genetic manipulation.

## MATERIALS AND METHODS

### Bacteria.

T. pallidum subsp. *pallidum* Nichols, provided by J.N. Miller (UCLA Geffen School of Medicine), was originally obtained from a patient with neurosyphilis in Baltimore, Maryland, in 1912 (44). The T. pallidum subsp. *pallidum* strains UW231B and UW249B isolated from the blood of neurosyphilis patients (45) were kindly provided by L.C. Tantalo, S.K. Sahi, and C.M. Marra at the University of Washington School of Medicine. T. pallidum subsp. *pallidum* SS14, T. pallidum subsp. *pallidum* Mexico A, T. pallidum subsp. *endemicum* Bosnia A, T. pallidum subsp. *pertenue* Gauthier, and T. pallidum subsp. *pertenue* Samoa F were obtained from D.L. Cox at the U.S. Centers of Disease Control and Prevention in Atlanta, Georgia.

### Sf1Ep cells.

T. pallidum was grown in a coculture system with Sf1Ep cottontail rabbit epithelial cells (NBL-11), a secondary cell culture obtained from the American Type Culture Collection (Baltimore, MD). These cells apparently underwent spontaneous transformation during passage in our laboratory and can be cultured indefinitely. Sf1Ep cells utilized in this study were at passage 19 to 60 unless otherwise noted (22). Sf1Ep cells were maintained as previously described (22) and were inoculated at 1 × 10^5^ cells per well or other indicated concentrations in 6-well tissue culture plates 24 hours prior to inoculation with T. pallidum.

### *In vitro* cultivation of T. pallidum.

T. pallidum was cultivated *in vitro* using TpCM-2 medium and the previously described procedure (22), unless otherwise indicated. TpCM-2 medium contains CMRL 1066 without phenol red, sodium bicarbonate, or l-glutamine (catalog no. C9500-03A; US Biological) as the basal medium. TpCM-1 was prepared as described previously (19) with the omission of CoCl_2_, cocarboxylase, catalase, and superoxide dismutase (which in our hands did not improve T. pallidum survival or growth). In some experiments, medium M199 without phenol red, sodium bicarbonate, or l-glutamine (M3769; Sigma-Aldrich) or equal parts of M199 and CMRL 1066 were utilized as the basal medium instead of CMRL 1066 to produce TpCM-3 and TpCM-4, respectively. Other chemical additives were of reagent grade and were obtained from Sigma-Aldrich. The T. pallidum culture media were prepared 24 hours prior to culture inoculation and were pre-equilibrated by drawing a vacuum in a Brewer jar (BBL GasPak) and refilling with 5% CO_2_:95% N_2_ four times. Vacuum was drawn a final time, and the Brewer jar was filled with 1.5% O_2_: 5% CO_2_: 93.5% N_2_. The medium was then placed in a tri-gas incubator (ThermoFisher Forma Model 3130 or HERAcell Vios 160i) at 1.5% O_2_, 5% CO_2_, and 93.5% N_2_ at 34°C overnight. Three hours prior to inoculation or passage, the medium in the 6-well tissue culture plates previously seeded with Sf1Ep cells was aspirated, and each well was rinsed with 1 ml TpCM-2 and then filled with 4 ml TpCM-2. The confluence of the Sf1Ep cells was subjectively estimated by comparing the percentage of the growth surface covered by attached cells. Typically, confluence is about 10% for Sf1Ep seeded 1 day prior to experiment initiation, although 5% to 20% is commonly observed. The plates were then pre-equilibrated as described above, placed in the low-oxygen incubator, and inoculated with T. pallidum freshly harvested from ongoing *in vitro* cultures or, in some instances, inoculated from frozen stocks (stored at −80°C in TpCM-2 + 15% glycerol) of either rabbit-propagated or *in vitro*-cultured T. pallidum. Multiplicity of infection (MOI) varied for each *in vitro* passage, typically ranging from ∼50 to 125 T. pallidum/Sf1Ep cell.

In experiments using frozen TpCM-2, freshly prepared TpCM-2 that had undergone overnight pre-equilibration in 1.5% O_2_: 5% CO_2_: 93.5% N_2_ was frozen at −20°C in tightly capped 15- or 50-ml polypropylene tubes. At the time of use, the caps of the tubes containing the frozen medium were loosened, and the frozen tubes were subjected to evacuation and refilling with the low O_2_ mixture before placement in the tri-gas incubator. After overnight thawing and equilibration, the medium can be used for preparation of the Sf1Ep cultures for T. pallidum inoculation as described above.

### T. pallidum quantification and data analysis.

Unless otherwise noted, experiments were performed using triplicate wells for three biological replicates per condition. T. pallidum motility and concentration were assessed as previously described using Helber Thoma counting chambers (Hawksley, Lancing, Sussex, UK) viewed under a darkfield microscope (22). Two counts were performed for each biological replicate and averaged. The data shown are the averages of the three biological replicates per condition ± SEM.
