# Supplementary material for: Parameters Affecting Continuous In Vitro Culture of Treponema pallidum Strains
Source: mBio. 2021 Feb 23;12(1):e03536-20. doi: 10.1128/mBio.03536-20 (PMC8545124; doi:10.1128/mBio.03536-20)
Supplement: TABLE S1 [file mbio.03536-20-st001.pdf]

**Table S1. P-values* for differences in generation times between *T. pallidum* strains**							
	**TPA NicholsA**	**TPA NicholsB**	**TPA Mexico A**	**TPA UW249B**	**TPA UW231B**	**TPA SS14**	**TEN Bosnia A**
**TPA NicholsA**		0.0835	0.0008	1.23×10^{-8}	1.60×10^{-10}	1.74×10^{-8}	7.96×10^{-6}
**TPA NicholsB**			0.0001	1.13×10^{-9}	1.70×10^{-11}	2.97×10^{-9}	7.96×10^{-6}
**TPA Mexico A**				0.0069	0.0004	0.0004	0.0304
**TPA UW249B**					0.4264	0.4931	0.7748
**TPA UW231B**						0.8731	0.3136
**TPA SS14**							0.3603

* Unequal variance, two-tailed. Significant p-values ($\leq$0.05) are shaded.
