# Supplementary figures and images for: Parameters Affecting Continuous In Vitro Culture of Treponema pallidum Strains
Source: mBio. 2021 Feb 23;12(1):e03536-20. doi: 10.1128/mBio.03536-20 (PMC8545124; doi:10.1128/mBio.03536-20)

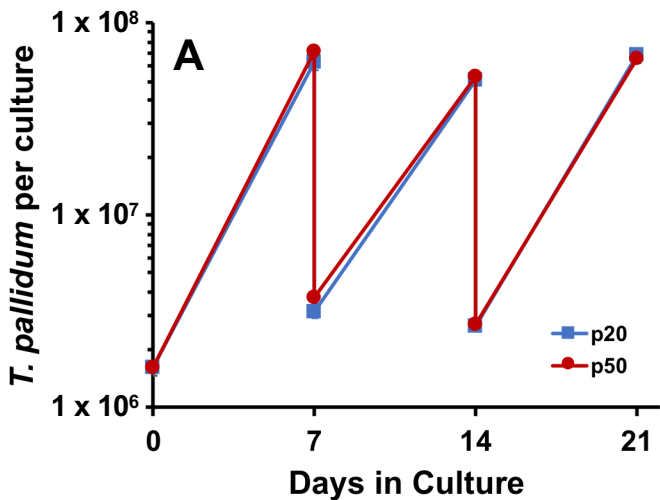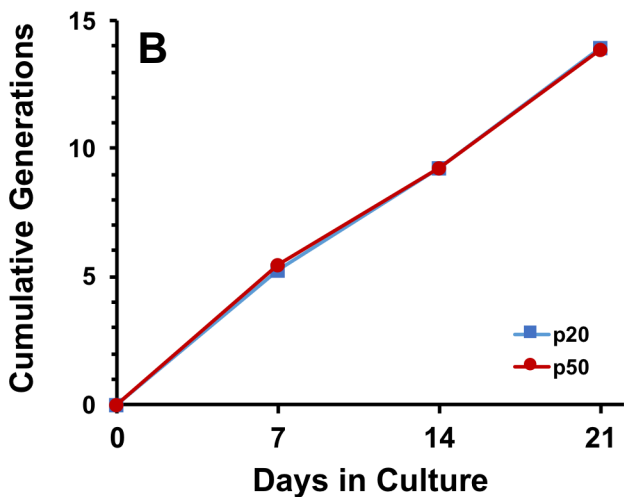

Supplement: FIG S1 [file mbio.03536-20-sf001.pdf]

# SS14

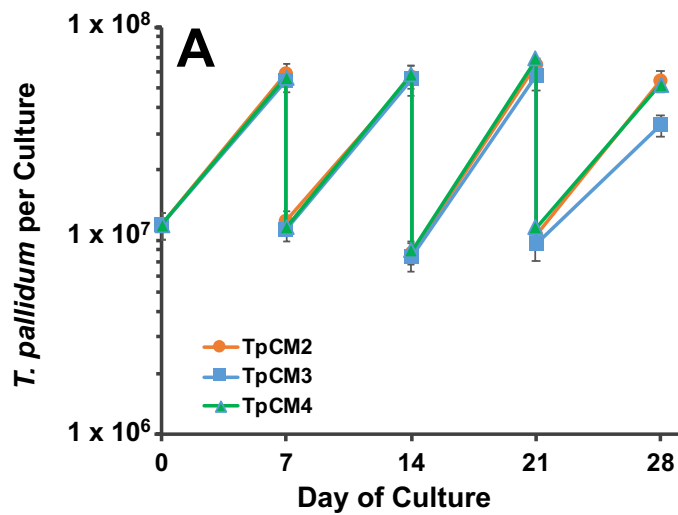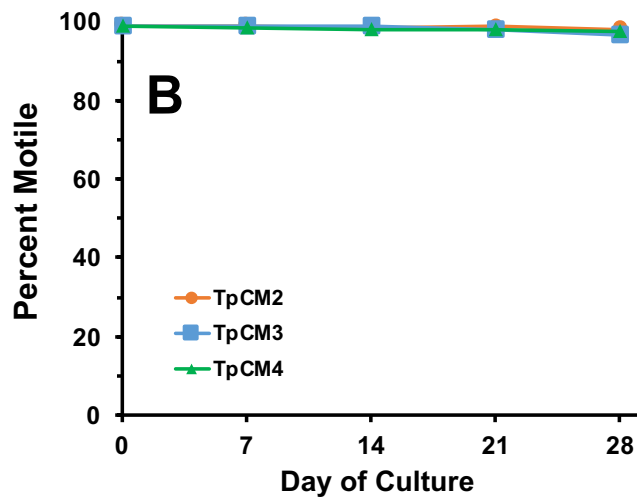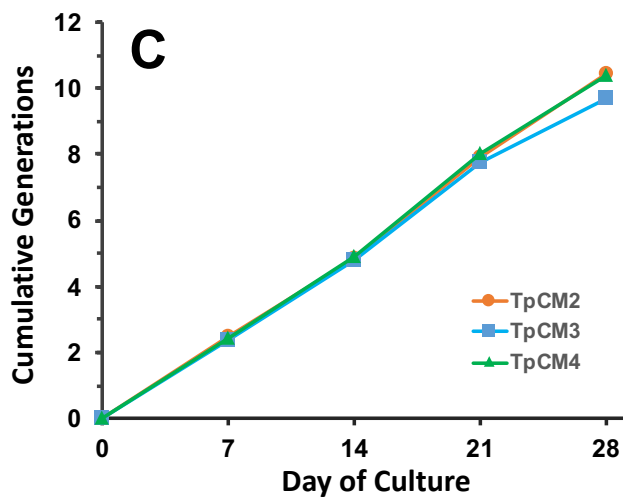

# UW231B

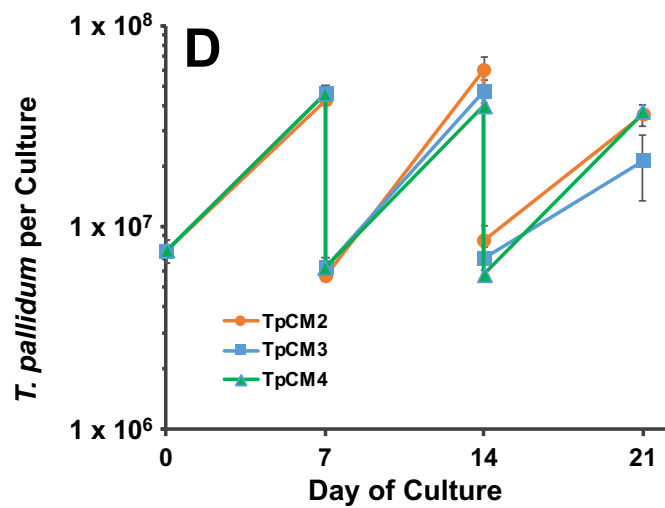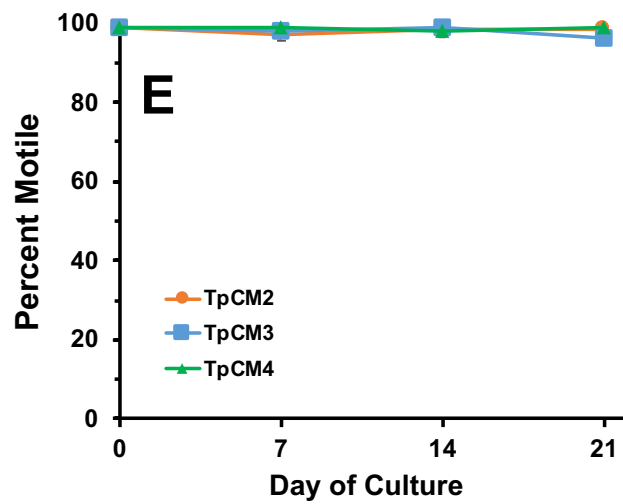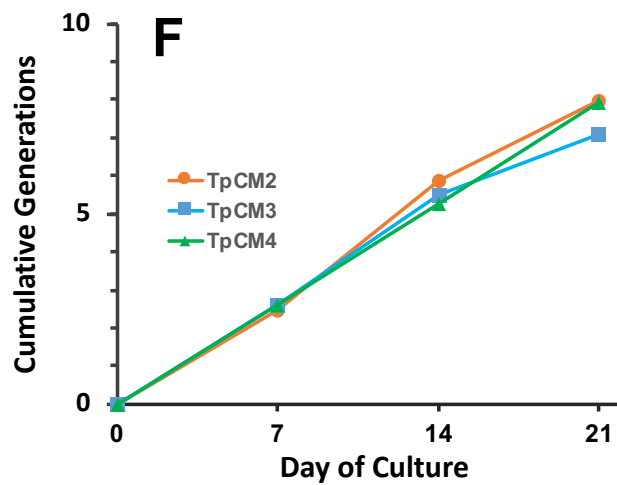

Supplement: FIG S2 [file mbio.03536-20-sf002.pdf]

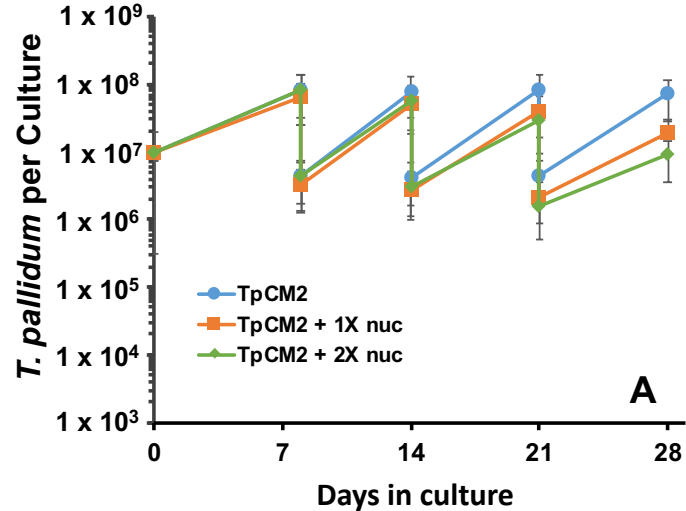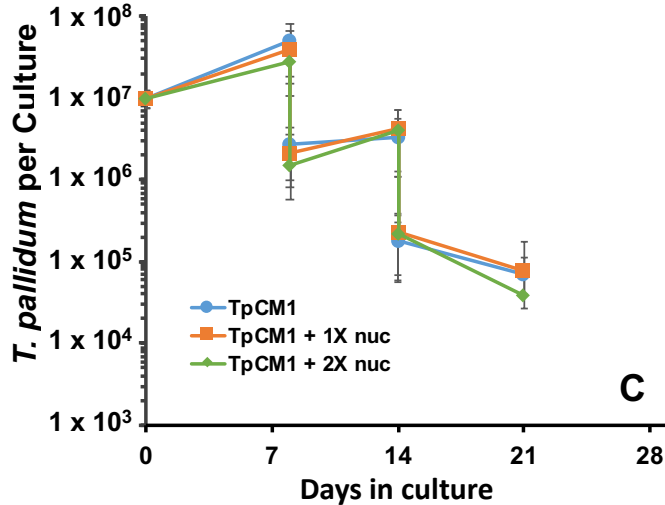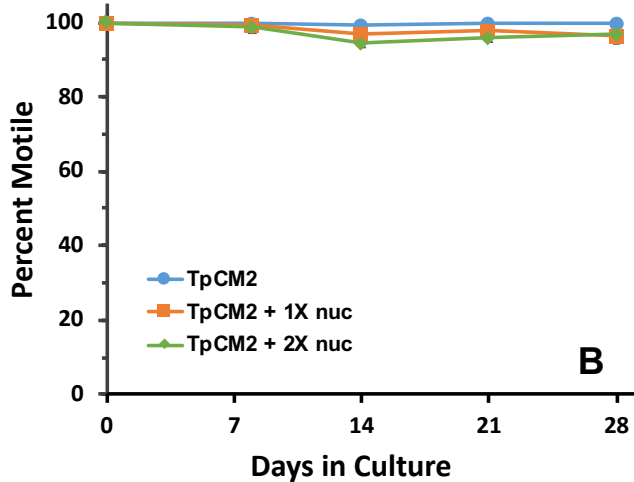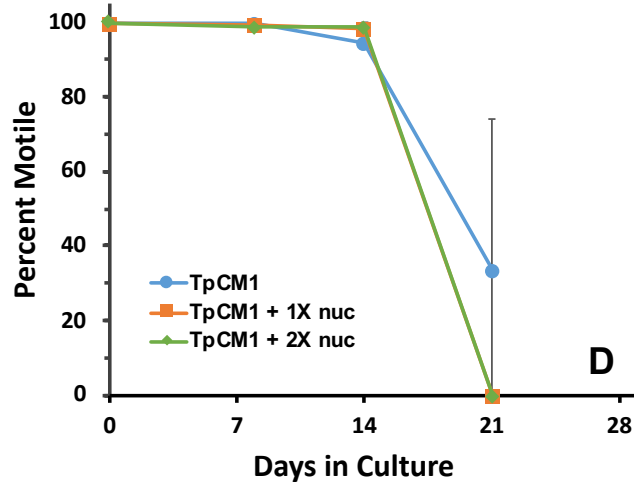

Supplement: FIG S4 [file mbio.03536-20-sf004.pdf]

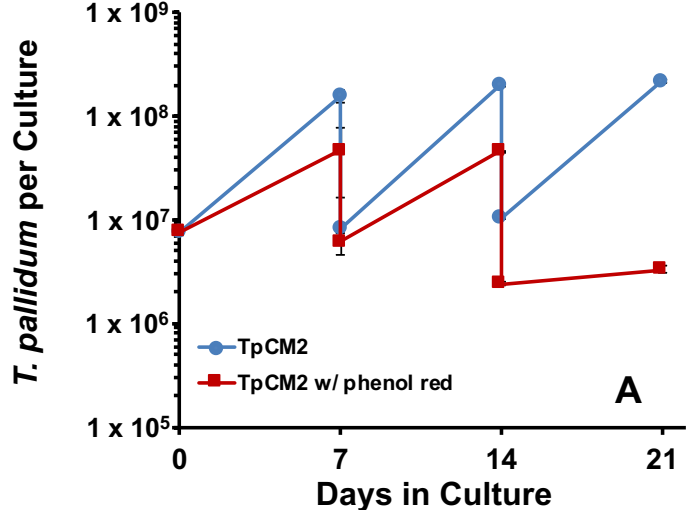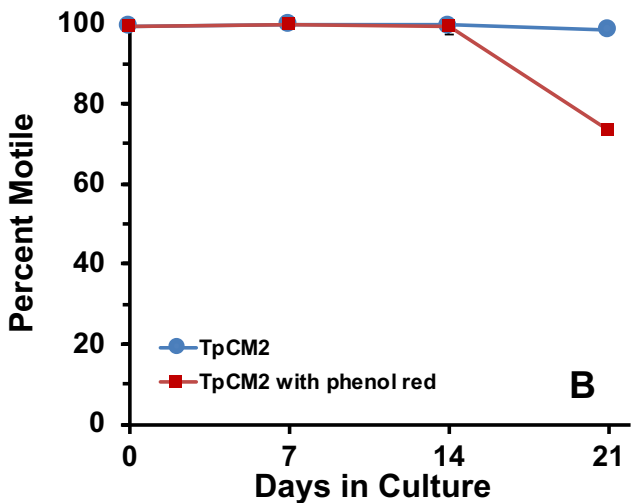

Supplement: FIG S5 [file mbio.03536-20-sf005.pdf]

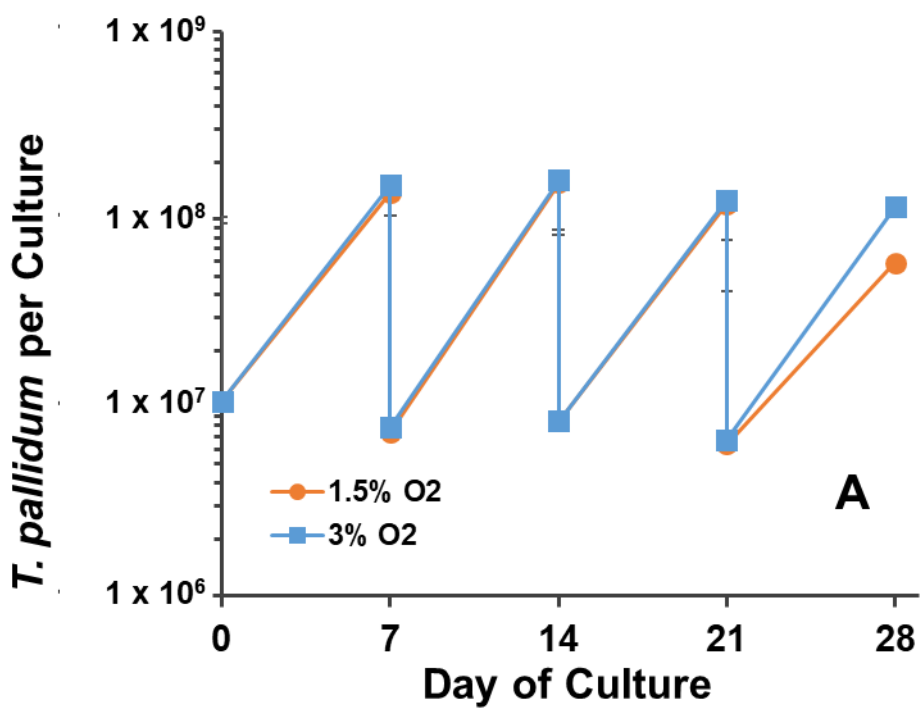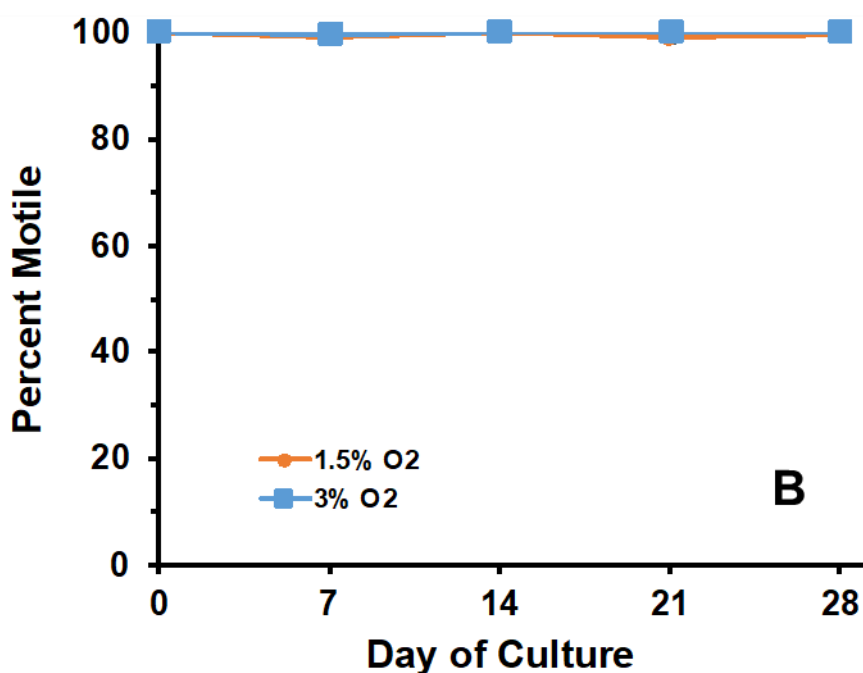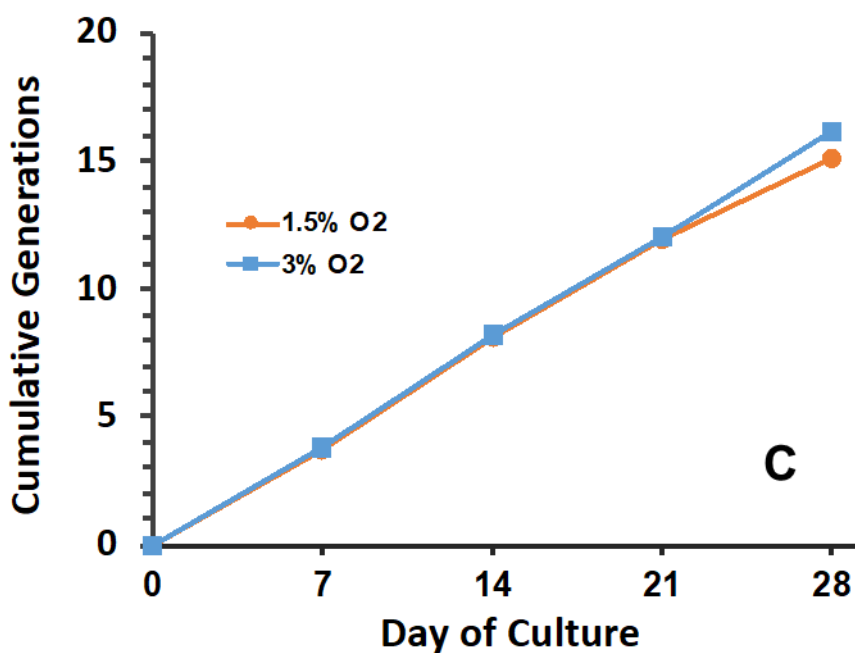

Supplement: FIG S6 [file mbio.03536-20-sf006.pdf]

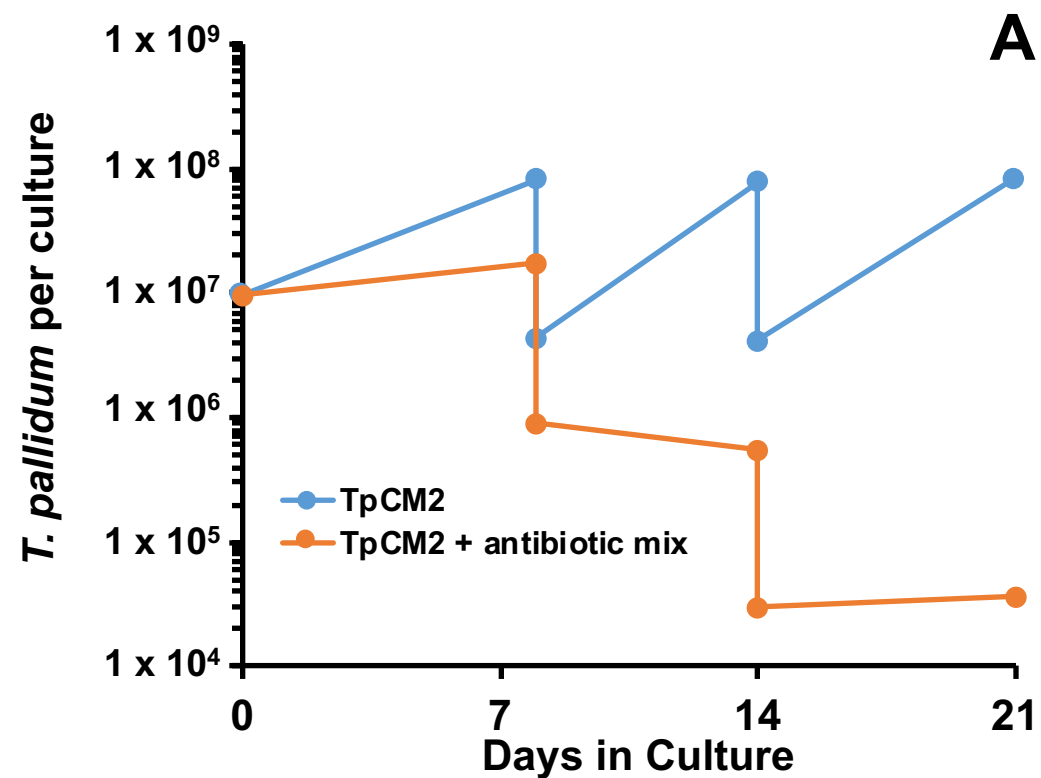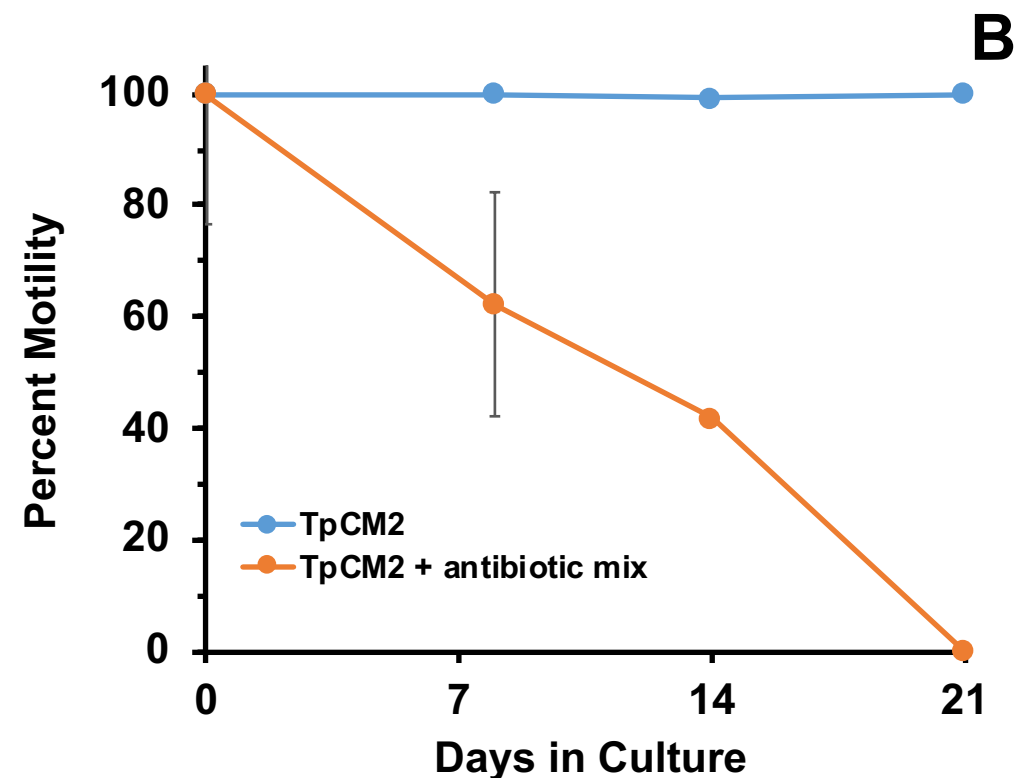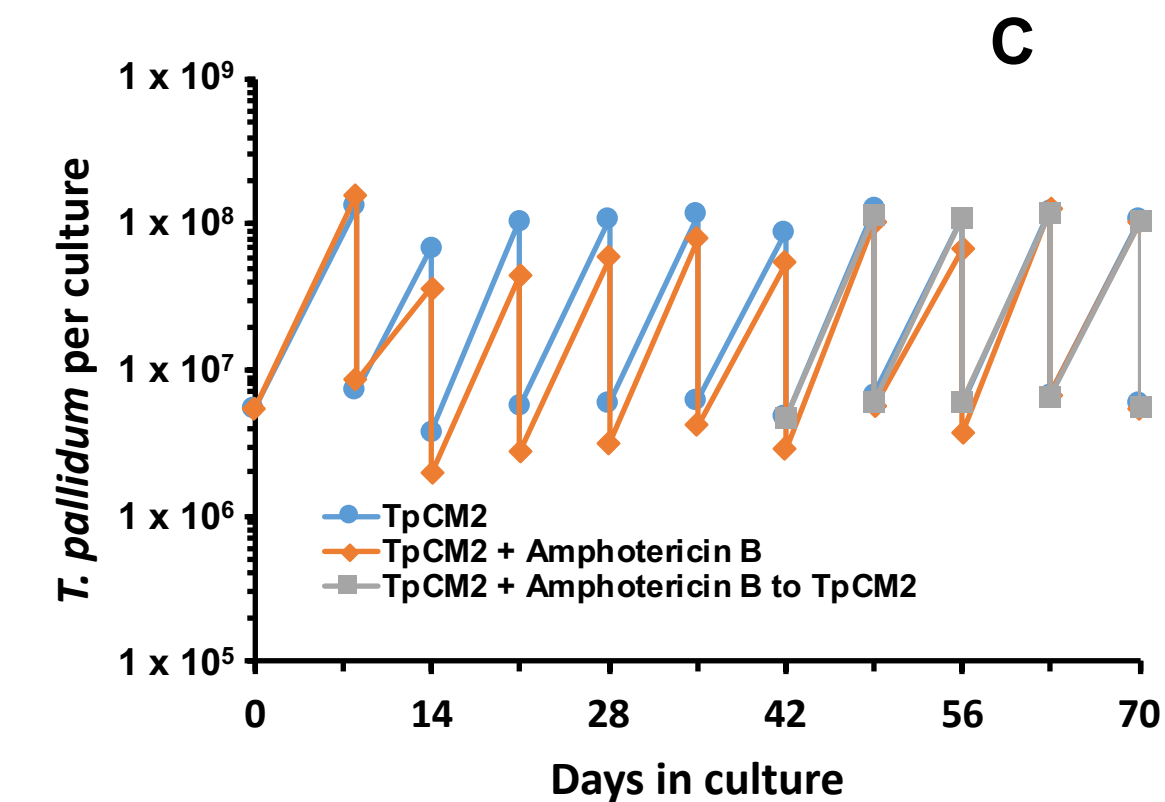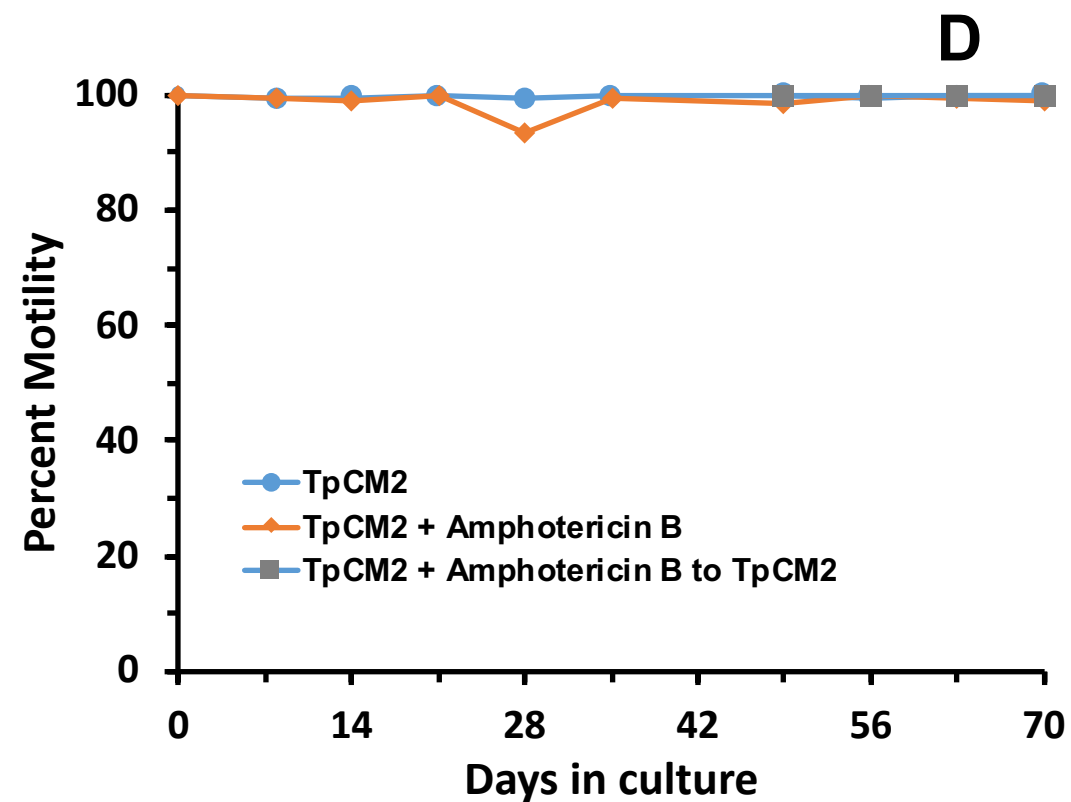

Supplement: FIG S7 [file mbio.03536-20-sf007.pdf]
