# Supplementary material for: Parameters Affecting Continuous In Vitro Culture of Treponema pallidum Strains
Source: mBio. 2021 Feb 23;12(1):e03536-20. doi: 10.1128/mBio.03536-20 (PMC8545124; doi:10.1128/mBio.03536-20)
Supplement: FIG S3 [file mbio.03536-20-sf003.pdf]

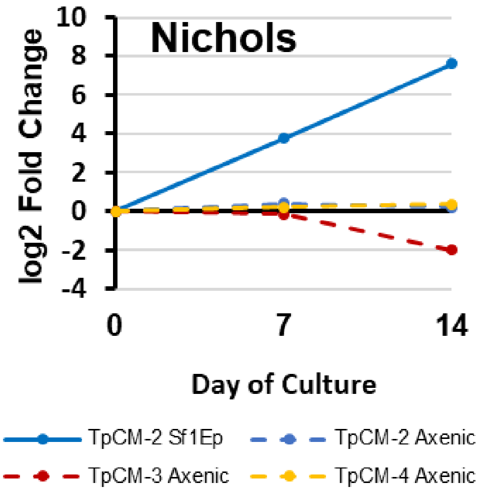
Nichols
log2 Fold Change
10
8
6
4
2
0
-2
-4
0
7
14
Day of Culture
TpCM-2 Sf1Ep
TpCM-2 Axenic
TpCM-3 Axenic
TpCM-4 Axenic

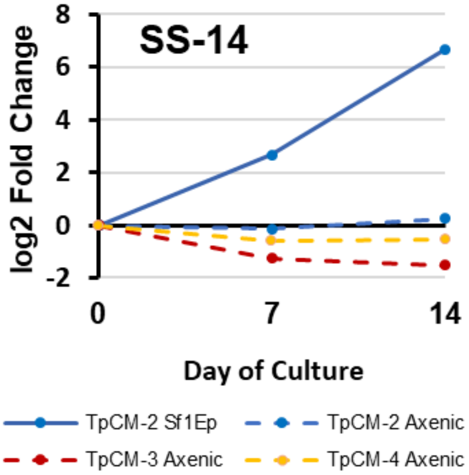
SS-14
log2 Fold Change
8
6
4
2
0
-2
0
7
14
Day of Culture
TpCM-2 Sf1Ep
TpCM-2 Axenic
TpCM-3 Axenic
TpCM-4 Axenic

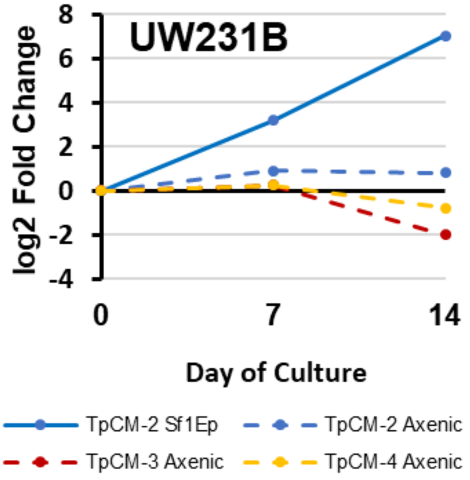
UW231B
log2 Fold Change
8
6
4
2
0
-2
-4
0
7
14
Day of Culture
TpCM-2 Sf1Ep
TpCM-2 Axenic
TpCM-3 Axenic
TpCM-4 Axenic
