# Supplementary material for: Parameters Affecting Continuous In Vitro Culture of Treponema pallidum Strains
Source: mBio. 2021 Feb 23;12(1):e03536-20. doi: 10.1128/mBio.03536-20 (PMC8545124; doi:10.1128/mBio.03536-20)
Supplement: TABLE S2 [file mbio.03536-20-st002.pdf]

**Table S2.** Components present in the 1000X nucleotide/nucleoside solution.		
**Component**	**Sigma catalog number**	**g/mL in 1000X solution**
2’ Deoxyadenosine	D1771	0.01
2’ Deoxycytidine HCl	D0776	0.0116
2’ Deoxyguanosine	D0901	0.01
Thymidine	T1895	0.01
Uridine 5’ triphosphate Na	U6750	0.001
Adenine	A2786	0.000106
Cytosine	C3506	0.000106
Guanine	G11950	0.000106
Thymine	T0895	0.000106
Uridine	U3003	0.000106
