# Supplementary material for: Parameters Affecting Continuous In Vitro Culture of Treponema pallidum Strains
Source: mBio. 2021 Feb 23;12(1):e03536-20. doi: 10.1128/mBio.03536-20 (PMC8545124; doi:10.1128/mBio.03536-20)
Supplement: TABLE S3 [file mbio.03536-20-st003.pdf]

Table S3. Components present in Eagle's MEM, and additional components present in CMRL 1066 and M199		
**Eagle's Minimal Essential Medium (MEM)**		
**Inorganic Salts:**	**Amino Acids:**<br>**(includes Nonessential AAs)**	**Vitamins:**
Calcium Chloride	L-Alanine	Choline Chloride
Magnesium Sulfate	L-Arginine	Folic Acid
Potassium Chloride	L-Aspartic Acid	Myo-Inositol
Sodium Chloride	L-Cystine	Niacinamide
Sodium Monophosphate	L-Glutamic Acid	D-Pantothenic Acid
**Other:**	Glycine	Pyridoxal
D-Glucose	L-Histidine	Riboflavin
	L-Isoleucine	Thiamine
	L-Leucine	
	L-Lysine	
	L-Methionine	
	L-Phenylalanine	
	L-Proline	
	L-Serine	
	L-Threonine	
	L-Tryptophan	
	L-Tyrosine	
	L-Valine	
**Additional Components - CMRL 1066 (23)**		
**Nucleic Acid Precursors:**	**Amino Acids:**	**Vitamins:**
2'-Deoxyadenosine	L-Cysteine	L-Ascorbic Acid
2'-Deoxyguanosine	Trans-4-Hydroxy-L-Proline	D-Biotin
2'-Deoxycytidine		Cocarboxylase
5-Methyldeoxycytidine		Coenzyme A
Thymidine		Flavin Adenine Dinucleotide
Uridine-5-Triphosphate		NAD
**Other:**		NADP
Cholesterol		Nicotinic Acid
D-Glucuronic Acid		PABA
Glutathione		Pyridoxine
Sodium Acetate		
Tween 80		
**Additional Components – M199 (24)**		
**Inorganic Salts:**	**Amino Acids:**	**Vitamins:**
Ferric Nitrate	L-Cysteine	L-Ascorbic Acid
Potassium monophosphate	Trans-4-Hydroxy-L-Proline	D-Biotin
**Nucleic Acid Precursors:**		Calciferol
Adenine Sulfate		Coenzyme A
Adenosine Triphosphate		Menadione
Adenosine Monophosphate		Nicotinic Acid
Deoxyribose		PABA
Guanine		Pyridoxine
Hypoxanthine		Retinol Acetate
**Other:**		DL-α-Tocopherol Phosphate
Cholesterol		
Glutathione		
Sodium Acetate		
Tween 80		
